# Protein kinase C-related kinase 1 and 2 play an essential role in thromboxane-mediated neoplastic responses in prostate cancer

**DOI:** 10.18632/oncotarget.4664

**Published:** 2015-07-20

**Authors:** Aine G. O'Sullivan, Eamon P. Mulvaney, Paula B. Hyland, B. Therese Kinsella

**Affiliations:** ^1^ UCD School of Biomolecular and Biomedical Sciences, UCD Conway Institute of Biomolecular and Biomedical Research, University College Dublin, Belfield, Dublin, Ireland

**Keywords:** thromboxane, receptor, cancer, prostate, protein kinase C-related kinase

## Abstract

The prostanoid thromboxane (TX) A_2_ is increasingly implicated in neoplastic progression, including prostate cancer (PCa). Mechanistically, we recently identified protein kinase C-related kinase (PRK) 1 as a functional interactant of both the TPα and TPβ isoforms of the human T prostanoid receptor (TP). The interaction with PRK1 was not only essential for TPα/TPβ-induced PCa cell migration but also enabled the TXA_2_-TP axis to induce phosphorylation of histone H3 at Thr11 (H3Thr11), an epigenetic marker both essential for and previously exclusively associated with androgen-induced chromatin remodelling and transcriptional activation. PRK1 is a member of a subfamily of three structurally related kinases comprising PRK1/PKNα, PRK2/PKNγ and PRK3/PKNβ that are widely yet differentially implicated in various cancers. Hence, focusing on the setting of prostate cancer, this study investigated whether TPα and/or TPβ might also complex with PRK2 and PRK3 to regulate their activity and neoplastic responses. While TPα and TPβ were found in immune complexes with PRK1, PRK2 and PRK3 to regulate their activation and signalling, they do so differentially and in a TP agonist-regulated manner dependent on the T-loop activation status of the PRKs but independent of their kinase activity. Furthermore, TXA_2_-mediated neoplastic responses in prostate adenocarcinoma PC-3 cells, including histone H3Thr11 phosphorylation, was found to occur through a PRK1- and PRK2-, but not PRK3-, dependent mechanism. Collectively, these data suggest that TXA_2_ acts as both a neoplastic and epigenetic regulator and provides a mechanistic explanation, at least in part, for the prophylactic benefits of Aspirin in reducing the risk of certain cancers.

## INTRODUCTION

The prostanoid thromboxane (TX) A_2_, synthesized from arachidonic acid by the sequential actions of cyclooxygenase (COX)-1/COX-2 and TXA_2_ synthase predominantly in platelets and macrophages, plays a central role within the vasculature dynamically regulating platelet activation status and vascular tone [1, 2]. TXA_2_ is also a potent *pro*-inflammatory and *pro*-mitogenic mediator promoting smooth muscle cell proliferation and restenosis in response to endothelial damage/vessel injury. In keeping with this, dysregulation of the levels of TXA_2_, its TXA_2_ synthase and its T prostanoid receptor (the TP) are implicated in a range of cardiovascular diseases [1, 2]. Hence, inhibiting TXA_2_ synthesis, action or both through use of Aspirin or TP antagonists is a key therapeutic strategy in the management of many diseases involving TXA_2_, including atherothrombosis and acute coronary syndrome.

By now, numerous studies have demonstrated increased expression of COX-1 and COX-2 and of their prostanoid metabolites in a range of cancers [3]. In support of this, several longitudinal studies show that long-term daily use of Aspirin reduces the risk of many prevalent cancers including of the colon, oesophagus, lung and prostate [4–6]. While such studies do not specify which COX-metabolite(s) are affected by Aspirin to account for its prophylactic benefits, particular attention has recently been directed to the role of TXA_2_, TXA_2_ synthase and its T prostanoid receptor/TP in various neoplasms [7]. For example, in addition to COX-2, increased levels of TXA_2_ and expression of TXA_2_ synthase and of the TP have been strongly correlated with bladder and colorectal cancer [8–10] and with non-small cell lung cancer, where TXA_2_ is the main tumour-promoting COX-2 metabolite [11–13]. Increased expression of COX-2, TXA_2_ synthase and of the TP has also been widely implicated in prostate cancer, with direct correlation of protein expression with Gleason score and pathologic stage [14, 15]. Within the cancer tissue, expression of TXA_2_ synthase and the TP localizes to areas of perineural invasion, a recognized mechanism whereby prostate cancer cells penetrate the prostatic capsule and spread to other tissues [7, 14].

In humans and other primates, TXA_2_ signals through two structurally related receptor isoforms, referred to as TPα and TPβ, that differ exclusively in their carboxyl-terminal (C)-tail domains [16]. While TPα and TPβ are encoded by the same gene (the TBXA2R), they are differentially expressed in a range of cell/tissue types and are under the transcriptional regulation of two distinct promoters designated Prm1 and Prm3, respectively [17–23]. Consistent with this, in the setting of prostate and breast cancer, the tumour suppressors Wilms' tumour (WT)-1 and hypermethylated in cancer (HIC)-1 bind to *cis*-elements within Prm1 to regulate TPα expression [24]. However, the factors regulating TPβ in those clinical settings remain to be identified. Functionally, as members of the G-protein coupled receptor (GPCR) superfamily, TPα and TPβ primarily couple to Gαq-phospholipase (PL) Cβ and to Gα_12_-RhoA activation [25, 26]. In addition, TPα and TPβ both lead to similar activation of the extracellular signal regulated protein kinase (ERK) and phosphatidylinositol 3′kinase (PI3′K) signalling cascades [27–29], but undergo distinct mechanisms of agonist-induced homologous [30, 31] and heterologous desensitization [32–34]. Hence, mechanistically, in the context of cancer, it is proposed that TP-mediated RhoA activation plays a critical role in the TXA_2_-induced tumour cell migration and metastasis while its ability to regulate the PI3′K and ERK cascades, including transactivation of the epidermal growth factor receptor (EGFR), may account for the influence of TXA_2_ on tumour cell proliferation/mitogenesis [7, 26–29].

Through additional mechanistic studies, we recently discovered that both TPα and TPβ directly interact with and regulate signalling by protein kinase C-related kinase (PRK) 1 [35], a RhoA effector widely implicated in prostate cancer. The PRKs, also referred to as protein kinase novel (PKN), constitute a subfamily of three structurally related yet functionally distinct Ser/Thr AGC kinases comprising PRK1/PKNα, PRK2/PKNγ and PRK3/PKNβ [36], downstream of RhoA and P13′K/PDK-1 signalling [37, 38]. However, while structurally related, the individual PRKs are functionally distinct and are each recognized as promising chemotherapeutic targets with several clinical trials underway, particularly for pancreatic and prostate cancers [39, 40]. For example, PRK2/PKNγ was recently implicated in triple negative breast cancer [41] and in controlling the migration and invasion of bladder tumour cells [42]. PRK3/PKNβ is required for invasive prostate cancer growth downstream of RhoC/PI3′K [43, 44] and Atu027, a *si*RNA-liposome complex targeting PRK3, has advanced to Phase II clinical trials for pancreatic cancer [40]. Due to its ability to directly interact with and be activated by the androgen receptor (AR), PRK1/PKNα has been strongly implicated in androgen-associated prostate cancer and in ovarian serous carcinoma [45–47]. In fact, by catalyzing histone H3 Thr11 phosphorylation in response to AR activation, PRK1 has been dubbed a “gate-keeper” of androgen-induced transcriptional activation and, therefore, of androgen-driven prostate cancer. Hence, PRK1 is now recognized as a key target in androgen-responsive prostate cancer, including in castrate-resistant prostate cancer (CRPC), the metastatic and lethal form of the disease that occurs following androgen deprivation therapy [47–49].

As stated, we recently identified PRK1 as a highly specific interactant of both TPα and TPβ [35]. Critically, the interaction was found to be functionally important not only in mediating TXA_2_-induced migratory responses but also revealed that, similar to that which occurs for the AR, signalling through PRK1 enables the TXA_2_-TP axis to induce chromatin remodeling (histone H3 Thr11 phosphorylation) in the prostate cancer PC-3 cell line and, in addition, to both mimic and augment androgen-induced responses in the LNCaP cell line [35]. This was the first demonstration that agents other than androgens, in this case TXA_2_, can induce histone H3 Thr11 phosphorylation, a PRK1-catalysed epigenetic marker essential for chromatin remodeling and transcriptional activation including in CRPC [47–49]. Therefore, given the increasing evidence of the link between elevated TXA_2_ signalling and TP expression with various cancers including, but not limited to prostate cancer, coupled with the recognized role of the Gα_12_-mediated RhoA/PRK1 signalling cascade in cancer, discovery of a direct, functional interaction between TPα and TPβ with PRK1 [35] provided an additional, previously unknown, mechanistic explanation for the “link between the COX-1/2 derived metabolite TXA_2_ and certain prevalent cancers”. However, that study was confined to PRK1 [35]. Hence, owing to the clear mechanistic and functional differences between the individual PRKs, be it PRK1/PKNα, PRK2/PKNγ and PRK3/PKNβ, combined with the increasing recognition of the importance of the TXA_2_-TP axis in various cancers, it was deemed imperative to investigate whether TPα and/or TPβ might also associate with and, thereby, regulate signalling by PRK2 or PRK3. Hence, focusing on the setting of prostate cancer, the aim of this study was to explore whether TPα and/or TPβ might also complex with and regulate the activity, neoplastic (proliferative and migratory) responses and, potentially, histone H3 Thr11 phosphorylation through PRK2 and PRK3. We reveal that while TPα and TPβ are found in functional complexes with PRK1, PRK2 and PRK3 to regulate PRK activation status and signalling, they do so differentially and in a TP agonist-regulated manner and dependent on the T-loop activation status of the PRKs but independent of their kinase activity. Furthermore, we show that TXA_2_-mediated neoplastic responses in prostate adenocarcinoma PC-3 cells, including histone H3 Thr11 phosphorylation, occurs through a PRK1- and PRK2-, but not PRK3-, dependent mechanism.

## RESULTS

### Association of PRK1, PRK2 and PRK3 with TPα and TPβ

**Figure 1:**
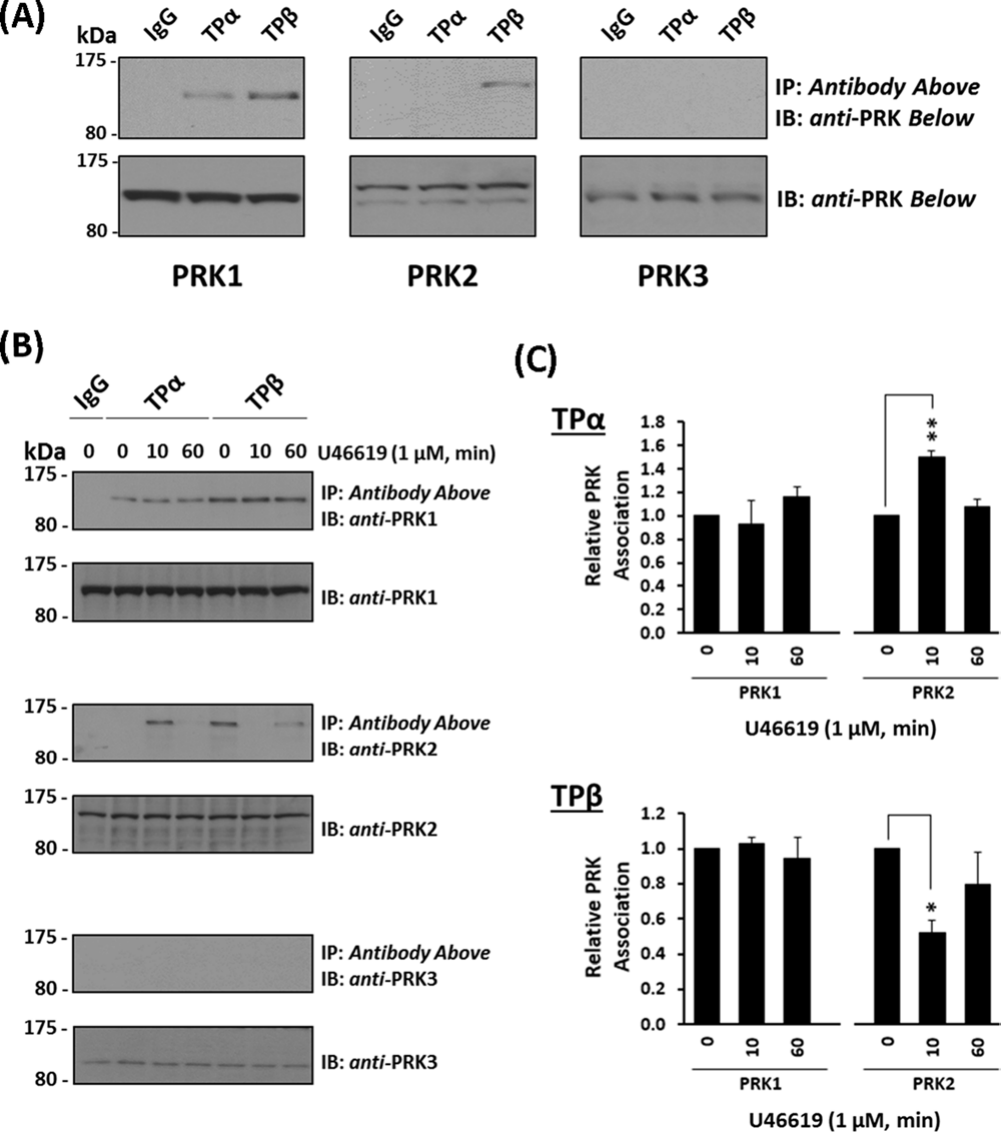
Association of PRK1 and PRK2 with TPα and TPβ in prostate PC-3 cells Panel **A.** PC-3 cells were immunoprecipitated with anti-TPα, anti-TPβ or, as controls, with the pre-immune (IgG) sera. Thereafter, immunoprecipitates (upper panels) or equivalent aliquots of whole cell lysates (20 μg/lane, lower panels) were immunoblotted (IB) with anti-PRK1, anti-PRK2 or anti-PRK3 antisera. The relative positions of the molecular size markers (kDa) are indicated to the left of the panels. Data shown are representative of at least three independent experiments (*n* ≥ 3). Panel **B.** PC-3 cells were incubated with U46619 (1 μM; 0–60 min) prior to immunoprecipitation with anti-TPα, anti-TPβ or, as controls, with the pre-immune (IgG) sera. Thereafter, immunoprecipitates (upper panels) or equivalent aliquots of whole cell lysates (20 μg/lane, lower panels) were IB with anti-PRK1, anti-PRK2 or anti-PRK3 antisera. Data *n* ≥ 3. Panel **C.** Bar charts show the mean relative levels of PRK1 or PRK2 associated with the anti-TPα or anti-TPβ immunoprecipitates, as determined by quantitative densitometry (± SEM), where levels associated with the respective immunoprecipitates in the absence of agonist are expressed as 1. The asterisks indicate where U46619 stimulation resulted in significant changes in complex-associated PRK1 or PRK2, where * and ** indicate *p* < 0.05 and *p* < 0.01, respectively.

PRK1 was recently identified as a highly specific and functionally important interacting partner of both TPα and TPβ in prostate cancer cells [35]. Hence, focusing on the setting of prostate cancer, the aim of this study was to investigate whether TPα and/or TPβ might associate with and regulate the activity, including neoplastic responses, through PRK2/PKNγ and PRK3/PKNβ, AGC kinases that like PRK1/PKNα act downstream of RhoA and PI3′K/PDK1 oncogenic signalling. To begin, the ability of PRK1, PRK2 or PRK3 to form complexes with TPα and TPβ in the metastatic prostate adenocarcinoma PC-3 cell line was investigated through co-immunoprecipitations using anti-TPα and anti-TPβ isoform-specific antibodies [26, 29]. Consistent with previous findings [35], PRK1 was detected in complexes with both TPα and TPβ, but not in control pre-immune IgG immunoprecipitates (Figure 1A). For PRK2, it was found in TPβ but not in TPα immune-complexes (Figure 1A). In contrast, PRK3 did not complex with either TPα or TPβ in PC-3 cells, despite its expression, albeit at lower levels than PRK1 or PRK2 (Figure 1A), and despite using increased protein input in the assays (Data not shown).

Thereafter, the influence of receptor activation on complex formation between the individual TPs and PRKs was investigated using the highly selective TP agonist U46619. Upon stimulation with U46619 for 0–60 min, levels of PRK1 associated with TPα and TPβ in complexes from PC-3 cells were not significantly altered relative to constitutive/basal levels, in the absence of agonist (Figure 1B & 1C). In contrast, the association of PRK2 with both TPα and TPβ was regulated in a time-dependent manner in response to U46619 (Figure 1B & 1C). In the absence of agonist, PRK2 was found in complex with TPβ, but not with TPα (Figure 1B & 1C). In response to U46619, PRK2 transiently complexed with TPα following 10 min stimulation, which diminished upon prolonged treatment for 60 min (Figure 1B & 1C). In contrast, while PRK2 complexed with TPβ in the absence of agonist, U46619 led to dissociation of the complex at 10 min, but at 60 min, levels of the TPβ:PRK2 complex were restored to that observed in the absence of agonist (Figure 1B & 1C). In the case of PRK3, it did not complex with TPα or TPβ in PC-3 cells either constitutively or following TP stimulation (Figure 1B).

**Figure 2:**
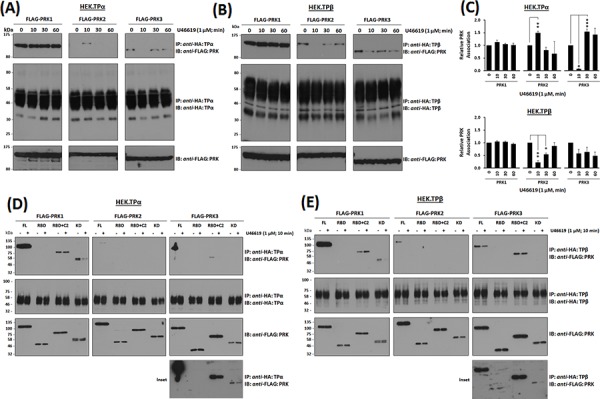
Association of PRK1, PRK2 and PRK3 with TPα and TPβ in HEK 293 cells Panels **A & B.** HEK 293 cells stably over-expressing HA-tagged TPα (Panel A) or TPβ (Panel B) and co-transfected with FLAG-tagged PRK1, PRK2 or PRK3 were incubated with U46619 (1 μM; 0–60 min) prior to immunoprecipitation with anti-HA antiserum. Immunoprecipitates (IP) were immunoblotted (IB) with anti-FLAG or anti-HA (upper and middle panels, respectively). To verify uniform expression of the PRKs, aliquots of the whole cell lysates (20 μg/lane) were IB with anti-FLAG antiserum (lower panels). Data *n* ≥ 4. Panel **C.** Bar charts show the mean relative levels of PRK1, PRK2 or PRK3 associated with the anti-HA immunoprecipitates, as determined by quantitative densitometry (± SEM), where levels in the absence of agonist are expressed as 1. The asterisks indicate where U46619 stimulation resulted in significant changes in complex-associated PRK1, PRK2 or PRK3, where *, ** and *** indicate *p* < 0.05, *p* < 0.01 and *p* < 0.001, respectively. Panels **D & E.** HEK 293 cells stably over-expressing HA-tagged TPα (Panel D) or TPβ (Panel E) and co-transfected with FLAG-tagged PRK1, PRK2 and PRK3 (FL, RBD, RBD+C2, kinase domain/KD) were incubated with U46619 (1 μM; 0–10 min) prior to immunoprecipitation with anti-HA antiserum and then immunoblotted (IB) with anti-FLAG or anti-HA (upper and middle panels, respectively). To verify uniform expression of the PRKs, aliquots of the whole cell lysates (20 μg/lane) were IB with anti-FLAG antiserum (lower panels). The inset panels show long duration exposures of the anti-FLAG-PRK3 immunoblots of the immunoprecipitates from HEK.TPα and HEK.TPβ cells. Data *n* ≥ 3.

To explore the possibility that the associations, or lack-of, between TPα and TPβ with the PRKs might be cell-type specific, TP:PRK complex formation was also examined in HEK293 cell lines that over-express TPα (HEK.TPα cells) or TPβ (HEK.TPβ cells) and the individual PRKs [33–35]. Consistent with findings in PC-3 cells, PRK1 strongly associated with both TPα and TPβ and the *nett*-association was not significantly altered in response to agonist-stimulation (Figure 2A, & 2C). Furthermore, while PRK2 did not associate constitutively with TPα, stimulation with U46619 for 10 min led to its transient association which was lost at 60 min post-stimulation (Figure 2A, & 2C), while the constitutive association of PRK2 with TPβ was lost at 10 min but restored at 60 min post-agonist stimulation (Figure 2A, & 2C). In the case of PRK3, unlike that which occurred in PC-3 cells, it complexed constitutively with both TPα and TPβ and in an agonist-regulated manner in the HEK293 cell lines. Specifically, the constitutive association of PRK3 with TPα was lost at 10 min but restored following U46619 stimulation for 30 min or 60 min (Figure 2A, & 2C). For TPβ, its constitutive association with PRK3 observed in the absence of agonist was largely unaffected following U46619-stimulation (Figure 2B, & 2C).

Structurally, the PRKs contain three highly conserved regions including an N-terminal Rho binding domain (RBD), a centrally located arachidonic acid-sensitive C2-like auto-inhibitory domain and a C-terminal catalytic kinase domain [36, 50]. To investigate whether the observed associations may involve direct association(s) between the TPs and PRKs, immune complexes generated between TPα and TPβ with the PRKs or with their corresponding RBD, RBD + C2 or kinase subdomains were investigated, including in response to short-term agonist-activation with U46619 (Figure 2D & 2E & Supplemental Figure 1). Consistent with previous findings [35], PRK1 associated with TPα and TPβ predominantly *via* its carboxy-terminal region incorporating its C2-like domain and catalytic kinase domain, while no association occurred with the N-terminal RBD. While the association of both TPα and TPβ with the full length PRK1 was largely unaffected by the activation status of the TPs, some differences were observed in their association with the C-terminal domains as a function of agonist stimulation (Figure 2D & 2E). For PRK2, consistent with findings in PC-3 cells (Figure 1B), while it did not associate with TPα under basal conditions, it did so in an agonist-dependent manner but, unlike that of PRK1, required expression of the entire PRK2 for association (Figure 2D). In contrast and consistent with findings in PC-3 cells (Figure 1B), PRK2 associated with TPβ in the absence of agonist through an association requiring the entire PRK2 protein but following short-term agonist stimulation, it dissociated from the TPβ complex (Figure 2E). In the case of PRK3, while it associated with TPα under constitutive/basal conditions through an association dependent, at least in part, on its C-terminal C2 and kinase domains, the interaction with the kinase domain was substantially weaker requiring extended exposures for detection and diminished in response to short-term agonist activation of TPα (Figure 2D & 2E, & Insets). Finally, although PRK3 associated with TPβ again through an association dependent on its C-terminal C2 and, in part, on its kinase domain, some differences were observed in their associations as a function of agonist stimulation. Hence, these data show that while both PRK1 and PRK3 may associate with TPα and TPβ via their C-terminal C2 and kinase domains, the association between the TPs with PRK2 requires the presence of the complete enzyme. Furthermore, the agonist-regulated interaction between TPα and TPβ with PRK1, PRK2 and PRK3 is both unique, but entirely consistent with previous data (Figure 1B, 2A & 2B).

Collectively, these data (Figure 1 & 2) confirm a constitutive association between PRK1 and TPα/TPβ which is not substantially regulated in response to receptor stimulation. In addition, while PRK2 may associate with TPβ, but not TPα, in the absence of agonist, both these associations are dynamically regulated in response to receptor activation. In the case of PRK3, while no complex was observed between TPα or TPβ in PC-3 cells, studies in HEK293 cells show that specific complexes can occur, and which are regulated in an agonist-dependent manner.

### Influence of TP agonist stimulation on the activation status of PRK1, PRK2 and PRK3

**Figure 3:**
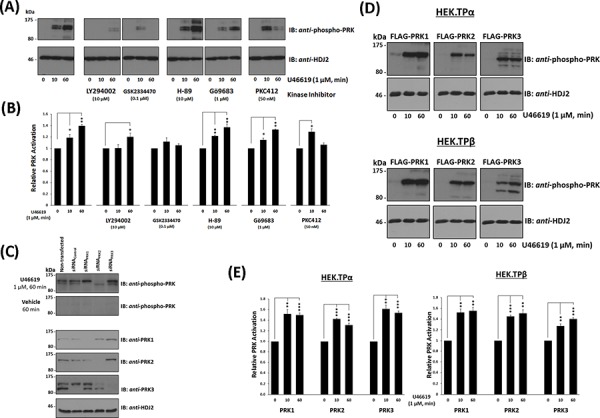
Influence of TP Agonist on the Activation of PRK1, PRK2 and PRK3 Panel **A.** PC-3 cells, serum-starved (0% FBS, 16 hr), were pre-incubated for 30 min with the listed protein kinase inhibitors or, as controls, with drug vehicle (0.001% DMSO) prior to stimulation with U46619 (1 μM) or vehicle (0.01% EtOH) for 0, 10 or 60 min. Cells were harvested and aliquots (20 μg/lane) were immunoblotted (IB) with anti-phospho-PRK1^Thr774^/PRK2^Thr816^/PRK3^Thr718^ (T-loop phosphorylation) and thereafter with anti-HDJ2 antisera to verify equal protein loading. Data *n* ≥ 3. Panel **B.** Bar charts show the mean relative activation levels of the PRK doublet species corresponding to PRK1 and PRK2, as determined by quantitative densitometry (± SEM), where levels in the absence of agonist are expressed as 1. The asterisks indicate where U46619 stimulation resulted in significant changes in activation, where * and ** indicate *p* < 0.05 and *p* < 0.01, respectively. Panel **C.** In order to identify the species of PRK subject to U46619-induced T-loop phosphorylation, PC-3 cells were initially transfected for 72 hr with 30 nM *si*RNA_PRK1_, *si*RNA_PRK2_, *si*RNA_PRK3_ or, as controls, with a scrambled *si*RNA_Control_. Thereafter, cells were serum starved and stimulated with U46619 for 60 min or with vehicle and then immunoblotted (20 μg/lane) with anti-phospho-PRK1^Thr774^/PRK2^Thr816^/PRK3^Thr718^ and with anti-PRK1, anti-PRK2, anti-PRK3 or, as loading controls, HDJ2 antisera, as indicated. Panel **D.** HEK.TPα or HEK.TPβ cells, transiently co-transfected with FLAG-tagged PRK1, PRK2 or PRK3 and serum-starved (0% FBS, 16 hr), were stimulated with U46619 (1 μM) or vehicle (0.01% EtOH) for 0, 10 or 60 min. Cells were harvested and aliquots (20 μg/lane) immunoblotted (IB) with anti-phospho-PRK1^Thr774^/PRK2^Thr816^/PRK3^Thr718^ and thereafter, to verify equal protein loading, with anti-HDJ2 antisera. Data *n* ≥ 3. Panel **E.** Bar charts show the mean relative activation levels of PRK1, PRK2 and PRK3 in HEK.TPα and HEK.TPβ cells, as determined by quantitative densitometry (± SEM), where levels in the absence of agonist are expressed as 1. The asterisks indicate where U46619 stimulation resulted in significant changes in activation, where ** and *** indicate *p* < 0.01 and *p* < 0.001, respectively.

In addition to directly associating with PRK1, agonist-activation of TPα and TPβ increases the activity of PRK1 [35]. The PRK family of RhoA effectors undergo activation through phosphoinositide-dependent kinase (PDK)-1 phosphorylation of a critical Thr within their kinase activation loops (T-loop phosphorylation) in response to upstream PI3′K/PDK-1 signalling [37, 38]. Hence, an anti-phospho-PRK1^Thr774^/PRK2^Thr816^/PRK3^Thr718^ antibody which recognises the phosphorylated, activated PRKs was used to monitor PRK1–3 activation status in PC-3 cells in response to stimulation with U46619. In the absence of agonist, no significant basal phosphorylation of the PRKs was detected (Figure 3A & 3B). Upon stimulation with U46619 for 10–60 min, there was a rapid appearance of a doublet band detected by the anti-phospho-PRK1^Thr774^/PRK2^Thr816^/PRK3^Thr718^, which co-migrated with species corresponding to the activated forms of PRK1 (lower band) and PRK2 (upper band) (Figure 3A & 3B). The identity of the PRKs and specificity of the phospho-PRK antibody was demonstrated through use of *silencing* (*si*)RNAs whereby specific knockdown of PRK1 and PRK2, but not PRK3, impaired detection of the U46619-induced phosphorylated PRK1 and PRK2 species, respectively (Figure 3C & Supplemental Figure 2A). In addition, all data were validated using at least two independent *si*RNAs to each of PRK1–3 (Supplemental Figure 2B). Thereafter, the involvement of the upstream kinases, PI3′K and PDK-1, in the TP-mediated activation of PRK1 and PRK2, was confirmed whereby inhibition of PI3′K and PDK-1, but not of the second messenger-regulated protein kinase (PK) A or PKC, impaired T-loop phosphorylation/activation of PRK1 and PRK2 in response to U46619 stimulation (Figure 3A & 3B). In addition, inclusion of the pan-PRK inhibitor PKC412 impaired the sustained T-loop phosphorylation/activation of PRK1 and PRK2 in response to U46619 stimulation (Figure 3A & 3B). Furthermore, U46619-induced PRK1 and PRK2 activation in PC-3 cells was both time and concentration dependent, where stimulation of cells with 250 nM U46619 for 60 min was found to be optimal (Supplemental Figure 3), while pre-incubation with the selective TP antagonist SQ29,548 inhibited the U46619-induced PRK activation further confirming TP specificity (Supplemental Figure 3).

While TP-mediated PRK3 activation did not occur in PC-3 cells, it was confirmed that the anti-phospho-PRK1^Thr774^/PRK2^Thr816^/PRK3^Thr718^ antibody can detect PRK3 when over-expressed in HEK293 cells (Supplemental Figure 4). Thereafter, TPα and/or TPβ-induced activation of PRK1, PRK2 and PRK3 was investigated in the HEK293 cell lines over-expressing TPα or TPβ and the individual PRKs. Consistent with findings in PC-3 cells, both PRK1 and PRK2 but also PRK3 underwent substantial T-loop phosphorylation in response to agonist-induced activation of TPα and TPβ (Figure 3D). Furthermore, inhibition of PI3′K and PDK-1 impaired T-loop phosphorylation of the PRKs in response to agonist-activation of both TPα and TPβ (Supplemental Figure 5).

Collectively, these data establish that agonist-activation of both TPα or TPβ leads to rapid and sustained T-loop phosphorylation of the PRK isozymes and which occurs through a PI3′K/PDK1-dependent mechanism.

### Role of the kinase activity and activation status of the PRKs on their association with TPα and TPβ

**Figure 4:**
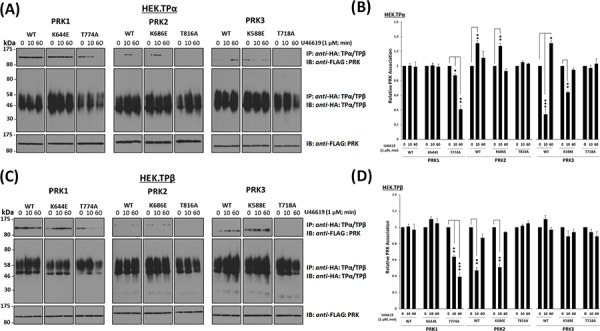
Influence of kinase activity and T-loop activation of PRK1, PRK2 and PRK3 on their agonist-regulated interaction with TPα and TPβ Panels **A & C.** HEK 293 cells stably over-expressing HA-tagged TPα (Panel A) or TPβ (Panel C) and co-transfected with FLAG-tagged wild type (WT), mutated kinase dead/dominant negative (K → E) or T-loop activation loop defective (T → A) forms of PRK1, PRK2 or PRK3 were incubated with U46619 (1 μM; 0–60 min) prior to immunoprecipitation with anti-HA antibody. Immunoprecipitates (IP) were immunoblotted (IB) with anti-FLAG or anti-HA (upper and middle panels, respectively). To verify uniform expression of the PRKs, aliquots of the whole cell lysates (20 μg/lane) were IB with anti-FLAG antibody (lower panels). Data *n* ≥ 3. Panels **B & D.** Bar charts show the mean relative levels of the PRKs associated with the anti-HA immunoprecipitates, as determined by quantitative densitometry (± SEM), where levels in the absence of agonist are expressed as 1. The asterisks indicate where U46619 stimulation resulted in significant changes in complex-associated PRK1, PRK2 or PRK3, where *, ** and *** indicate *p* < 0.05, *p* < 0.01 and *p* < 0.001, respectively.

Findings herein have established that the association and T-loop activation status of the PRKs with TPα/TPβ are dynamically regulated in response to agonist-induced receptor activation. Hence, it was next sought to investigate whether the activity and/or the activation status of the PRKs may play a role in their respective agonist-regulated associations with TPα and TPβ. For this end, the ability of either the wild-type, kinase-defective (PRK1^K644E^, PRK2^K686E^, PRK3^K588E^), or T-loop phosphorylation-defective (PRK1^T774A^, PRK2^T816A^, PRK3^T718A^) forms of PRK1–3 to form complexes with either TPα or TPβ was investigated in HEK.TPα and HEK.TPβ cells, respectively. The level and pattern of agonist-regulated associations of each of the kinase-defective PRK variants with either TPα or TPβ was not significantly different from those of the wild-type PRK1–3 (Figure 4A–4D). In the case of the T-loop phosphorylation defective PRK variants, in the absence of agonist, PRK1^T774A^ was capable of associating with TPα and TPβ (Figure 4A & 4C; T774A). However, in response to U46619 stimulation, the association of PRK1^T774A^ with both TPα and TPβ was lost/diminished in a time-dependent manner, being decreased at 10 min and further reduced or absent following prolonged stimulation (Figure 4A & 4C; T774A). Furthermore, neither PRK2^T816A^ nor PRK3^T718A^ associated with TPα or TPβ complexes, either in the absence of agonist or upon TP stimulation (Figure 4A & 4C; T816A & T718A).

Taken together, these data indicate that while the kinase activity *per se* does not influence the association of the PRKs with TPα/TPβ, their T-loop phosphorylation, a measure of their activation status, is critical in determining the ability of the PRKs to associate or complex with TPα and TPβ. In addition, while previous reports [35] indicated that the TP:PRK1 association was not regulated in response to TP stimulation, data herein involving the T-loop phosphorylation-defective PRK1^T774A^ suggest that sustained association of PRK1 with both TPα and TPβ is dependent on its activation status.

### Influence of TP agonist- activation on PRK1/2/3-mediated PC-3 cell proliferation and migration

The PRK isozymes each play an essential role in neoplastic processes, including in cancer cell proliferation, migration and invasion [42, 43]. In addition, disruption of PRK1 expression impairs TP-mediated PC-3 cell migration [35]. Hence, herein, the influence of the individual PRKs on TP-mediated prostate cancer cell responses, including cell proliferation, anchorage-independent growth (colony formation) and migration was investigated.

Stimulation with U46619 led to time- and concentration-dependent increases in proliferation, colony formation and migration of PC-3 cells with optimal responses occurring at 10–50 nM U46619 in all cases (Supplemental Figure 6). Furthermore, the specificity that U46619 mediated these effects through the TP was confirmed whereby the selective TP antagonist SQ29,548 blocked each of these U46619-induced responses in PC-3 cells (Supplemental Figure 6).

**Figure 5:**
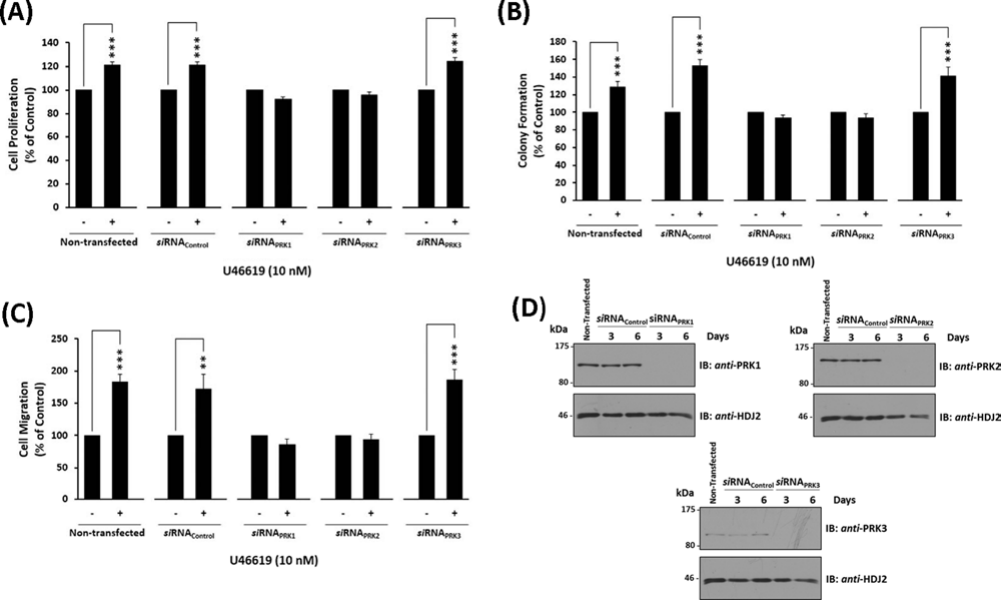
Effect of siRNA-mediated disruption of PRK1, PRK2 and PRK3 expression on TP agonist-induced proliferation and migration of PC-3 cells Panels **A-D.** PC-3 cells were transfected for 48 hr with 30 nM *si*RNA_PRK1_, *si*RNA_PRK2_, *si*RNA_PRK3_ or, as controls, with a scrambled *si*RNA (*si*RNA_Control_) prior to stimulation with U46619 (10 nM) or vehicle (0.0001% EtOH) for the indicated time specific to the assay, where non-transfected cells served as a reference. Panel A: For analysis of proliferation, 72 hr post-*si*RNA transfection PC-3 cells were stimulated for 48 hr with U46619 (10 nM) or vehicle (0.0001% EtOH). Panel B: For analysis of colony formation, 48 hr post-*si*RNA transfection PC-3 cells were stimulated with U46619 (10 nM) or vehicle (0.0001% EtOH) in soft agar and assessed 10 day post-treatment for colony formation. Panel C: For analysis of migration, 72 hr post-*si*RNA transfection PC-3 cells were stimulated for 8 hr with U46619 (10 nM) or vehicle (0.0001% EtOH). In Panels A-C, the bar charts show mean relative levels of PC-3 cell proliferation, colony formation and migration (± SEM, *n* ≥ 3), where levels in the vehicle-treated cells are assigned *a* value of 100%. The asterisks indicate where U46619-stimulation resulted in significant increases in proliferation, colony formation or migration by PC-3 cells compared to vehicle-treated cells, where ** and *** indicates *p* < 0.01 and *p* < 0.001, respectively. Panel D: Validation of the specificity and sustained *si*RNA-mediated disruption of PRK1, PRK2 and PRK3 expression was confirmed by immunoblot analysis of whole cell lysates (20 μg/lane) with the respective anti-PRK1/2/3 or, to validate protein loading, with anti-HDJ2 antisera where analysis was carried out 3 and 6 day post-transfection. Data *n* ≥ 3.

To determine the role of the individual PRKs on TP-mediated prostate cancer cell responses, the effect of *si*RNA-targeted disruption of the individual PRKs on U46619-induced responses in PC-3 cells was examined. Specific s*i*RNA-disruption of both PRK1 and PRK2 expression in PC-3 cells significantly impaired U46619-induced cell proliferation, colony formation and cell migration, while *si*RNA-disruption of PRK3 or control scrambled *si*RNA sequences had no effect on these U46619-induced responses (Figures 5A–5C). The specificity of the individual *si*RNA sequences to specifically disrupt PRK1, PRK2 and PRK3 expression, and for prolonged periods (up to 6 day post-transfection), was confirmed through western blotting (Figure 5D & Supplemental Figure 2).

### Influence of TP agonist- activation on PRK1/2/3-mediated Histone H3Thr11 phosphorylation

It has been recently established that agonist-activation of TPα and TPβ can, in turn, lead to PRK1-catalysed H3Thr11 phosphorylation in prostate cancer cells, representing the first demonstration that agents other than androgens can induce this key epigenetic modification and recognized gate-keeper of androgen-induced transcriptional activation [35, 47]. Hence, given our demonstration that the TPs may also associate with and modulate the activity and downstream signalling of the PRKs, including neoplastic responses, it was sought to explore whether TP-mediated activation of PRK2 and/or PRK3 may also lead to phosphorylation of H3Thr11 in PC-3 cells. U46619-induced H3Thr11 phosphorylation was analysed using a specific anti-phospho-H3Thr11 antibody, where PC-3 cells growth-arrested in metaphase with colcemid served as a positive control for H3Thr11 phosphorylation (Supplemental Figure 7A). Consistent with previous data (Supplemental Figure 6), 50 nM U46619 was the optimal agonist concentration required to induce H3Thr11 phosphorylation through the TPs (Supplemental Figure 7A). Furthermore, stimulation of PC-3 cells with 50 nM U46619 led to a time-dependent increase in H3Thr11 phosphorylation and the selective TP antagonist SQ29,548 specifically inhibited the U46619-induced H3Thr11 phosphorylation, confirming TP specificity (Supplemental Figure 7B). In addition, treatment with the vehicle alone did not bring about an increase in H3Thr11 phosphorylation (Supplemental Figure 7B).

**Figure 6:**
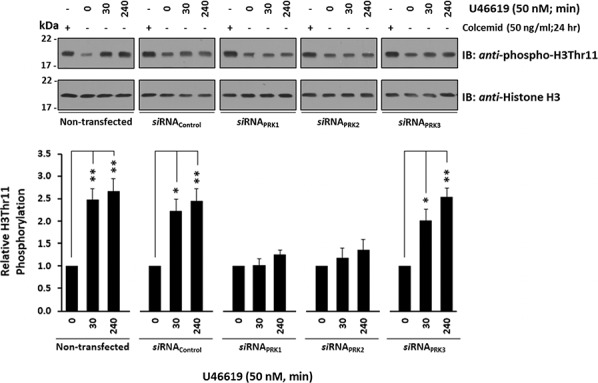
Effect of TP agonist stimulation on PRK-mediated H3Thr11 phosphorylation in PC-3 cells PC-3 cells were transfected for 72 hr with 30 nM *si*RNA_PRK1_, *si*RNA_PRK2_, *si*RNA_PRK3_ or, as controls, with a scrambled *si*RNA (*si*RNA_Control_) prior to stimulation with U46619 (50 nM; 0–240 min) or vehicle (0.0005% EtOH), where non-transfected cells served as a reference. As a positive reference for H3Thr11 phosphorylation, cells were growth-arrested by treatment with colcemid (50 ng/ml) for 24 hr. In all cases, extracted histones were immunoblotted (IB) with anti-phospho-H3Thr11 (upper panels) and back-blotted with anti-histone H3 (lower panels) antisera. The bar charts show the mean relative levels of H3Thr11 phosphorylation relative to total histone H3 levels, as determined by densitometry (± SEM, *n* ≥ 3), where levels in the vehicle-treated cells were assigned *a* value of 1. The asterisks indicate where U46619 stimulation resulted in significant changes in the levels of H3Thr11 phosphorylation, where * and ** indicate *p* < 0.05 and *p* < 0.01, respectively.

Specific s*i*RNA-disruption of both PRK1 and PRK2 expression in PC-3 cells significantly impaired U46619-induced H3Thr11 phosphorylation, while *si*RNA-disruption of PRK3 or *si*RNA scrambled control sequences had no effect (Figure 6). In all cases, rescreening of the anti-phospho-H3Thr11 blots with anti-histone H3 antibody confirmed that any differences in H3Thr11 phosphorylation were not due to discrepancies in histone H3 levels (Figure 6).

### Role of PRK1 on the Association of PRK2 with TPα and TPβ in PC-3 cells

**Figure 7:**
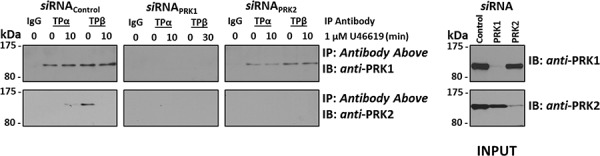
Effect of PRK1 on the Association of PRK2 with TPα and TPβ in PC-3 cells PC-3 cells were transfected for 72 hr with 30 nM *si*RNA_PRK1_, *si*RNA_PRK2_ or, as controls, with a scrambled *si*RNA (*si*RNA_Control_) prior to stimulation with U46619 (1 μM; 10 min) or vehicle (0.01% EtOH), and then immunoprecipitated with anti-TPα, anti-TPβ or, as controls, with the pre-immune (IgG) sera. Thereafter, immunoprecipitates (upper panels) or equivalent aliquots of whole cell lysates (20 μg/lane, lower panels) were IB with anti-PRK1 or anti-PRK2 antisera. Data *n* ≥ 3.

Collectively, data herein show a strong dependence on PRK1 and PRK2, but not on PRK3, in mediating TP-induced neoplastic responses, including cell proliferation, migration and H3Thr11 phosphorylation, in PC-3 cells. Moreover, *si*RNA-targeted disruption studies showed near-on equivalent dependence on either PRK1 or PRK2 in inducing these TP-mediated responses, despite substantial differences in the levels of PRK1 *versus* PRK2 expression in PC-3 cells and despite clear differences in their agonist-regulated associations with TPα and TPβ (Figure 1). Due to these findings, it was sought to establish whether expression of PRK1 in PC-3 cells might influence the association of TPα/TPβ with PRK2 or *vice versa*. Hence, the effect of *si*RNA-mediated disruption of PRK1 and PRK2 on their ability to complex with TPα and TPβ in the absence or presence of U46619 (10 min) stimulation was investigated through co-immunoprecipitations from PC-3 cells where cells transfected with scrambled *si*RNA sequences served as controls (Figure 7). Notably, upon *si*RNA-disruption of PRK1 expression, PRK2 was not found to associate with TPα or TPβ either in the presence or absence of TP agonist (Figure 7). However, upon knockdown of PRK2 expression, levels of PRK1 complexed with TPα or TPβ were not significantly affected and were comparable to control levels in the presence of *si*RNA_Control_ (Figure 7). These data suggest that the association that exists between PRK1 with either TPα or TPβ is independent of the expression of and/or potential association with PRK2. However, these data also suggest that expression and/or prior association of PRK1 with TPα and TPβ may be a prerequisite for association of PRK2 with the TPs. Specifically, both the agonist-dependent association of PRK2 with TPα or the constitutive interaction of PRK2 with TPβ is dependent on the expression of PRK1 and/or on the association of PRK1 in complex with either TPα or TPβ.

## DISCUSSION

Androgen deprivation therapy (ADT) remains the principal treatment for patients with locally advanced, relapsed or metastatic prostate cancer [48]. Despite initial success, many men eventually fail this therapy and metastatic prostate cancer develops to the final castrate-resistant stage, for which no cure is available and only palliative therapy can be given [51]. Hence, increased understanding of the mechanisms that underlie CRPC is needed to develop novel therapeutic approaches for this disease [48]. Although the AR can remain functional and continue to be activated during CRPC, other androgen-independent signalling pathways also play a key role including disturbed growth factor signalling and survival pathways [51]. Key among the findings which may account for the aberrant activation of the AR in prostate cancer was the discovery that, in addition to classical ligand-binding mechanisms, transcriptional activity of the AR is controlled through its direct binding to the RhoA effectors PRK1 and PRK2 [46]. In fact, as stated, by catalysing H3Thr11 phosphorylation in response to androgen signalling, PRK1 functions as a gatekeeper of AR-regulated gene expression and, in turn, of deleterious growth of prostate cancer [47]. Moreover, PRK1 can control the transcriptional activation of AR-target genes even in the absence of testicular androgens or in the presence of anti-androgens, highlighting a role for PRK1 in prostate tumour growth and spread which occurs during ADT and CRPC [46].

There is significant evidence to support the role of the COX-1/2-derived TXA_2_ in tumour progression and metastasis, including within the prostate [7, 14, 52]. Mechanistically, TP-mediated RhoA activation plays a critical role in the TXA_2_-induced tumour cell migration and metastasis while its ability to regulate the PI3′K and ERK cascades may account for the influence of TXA_2_ on tumour cell proliferation/mitogenesis [7, 26–29]. In addition, we recently discovered a novel signalling pathway in prostate tumour cells involving the direct interaction of both TPα and TPβ with PRK1 [35]. Critically, this discovery revealed that agonist-stimulation of TPα/TPβ can directly activate PRK1 and established for the first time that agents other than androgens can induce chromatin remodelling through PRK1- mediated phosphorylation of histone H3 at Thr11 [35]. Moreover, it was established that TXA_2_-mediated PRK1 signalling can both mimic and augment androgen-induced H3Thr11 phosphorylation and cell migration in prostate cancer, providing a previously unknown functional link between the TXA_2_- TPα/TPβ signalling axis and the AR [35].

As stated, PRK1/PKNα, along with PRK2/PKNγ and PRK3/PKNβ, are members of a family of structurally related, yet functionally distinct Ser/Thr AGC kinases which, like PRK1, are strongly implicated in tumour promotion including within but not limited to the prostate [42, 43, 46]. Hence, focusing within the setting of prostate cancer, we sought to investigate whether, like PRK1, PRK2 and PRK3 might also associate with TPα and/or TPβ to mediate TXA_2_-induced chromatin remodelling and cell growth/metastasis, thereby gaining a greater mechanistic insight into how the TXA_2-_TP signalling axis, and target of Aspirin, can contribute to prostate tumorigenesis. In our study, we provide evidence that TPα and TPβ can form complexes/associate with all three of the PRK isozymes, but that the association occurs in a cell-specific manner whereby PRK1 and PRK2 can both associate with TPα/TPβ endogenously expressed in the prostate cancer PC-3 cell line, while PRK3 only associates with TPα/TPβ when over-expressed in HEK293 cells. However, the associations between TPα/TPβ with both PRK2 and PRK3 are both structurally distinct and highly regulated in response to agonist-stimulation of the TPs. Studies with the individual subdomains of the PRKs suggest that the associations with PRK1 and PRK3, either in the absence or presence of agonist, are predominantly dependent on their C-terminal C2 and kinase domains, but the association with PRK2 requires the presence of the complete enzyme for complex formation with TPα/TPβ. The molecular basis of the differential associations between the TPs and PRKs is currently unknown and will require additional investigations, such as through detailed biophysical studies of the complexes to define/identify the main structural motifs/residues involved.

**Figure 8:**
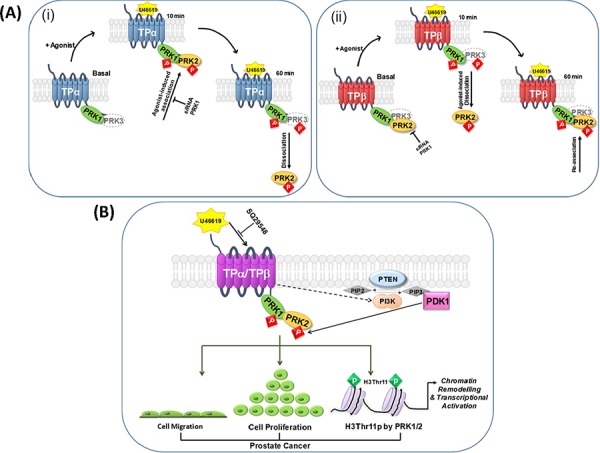
Proposed model of the association of TPα/TPβ with PRK1-3, and the implications for prostate tumour progression Panel **A.** In the prostate carcinoma PC-3 cell line, under constitutive/basal conditions PRK1 is recruited into a complex with both the TPα *(i)* and TPβ *(ii)* isoforms while PRK2 distinctly forms a complex only with that of TPβ. Following short-term agonist (U46619)-stimulation of TPα/TPβ (10 min), whereas there is no measurable alteration in the level of PRK1 associated with either TP isoform, a change occurs whereby PRK2 dissociates from TPβ *(ii)*, while transiently associating into a complex with TPα *(i)*. Following prolonged agonist-activation of TPα/TPβ (60 min), PRK2 dissociates from TPα *(i)* and is recruited once more into a complex with TPβ *(ii)*. Hence, the PRK:TPα *(i)* and PRK:TPβ *(ii)* complexes observed at 60 min post-agonist mimic those found basally, in the absence of agonist. Although not found to occur in PC-3 cells, PRK3 also has the ability to associate with TPα *(i)* and TPβ *(ii)*, such-as when co-expressed in HEK293 cells. In response to receptor stimulation, while there is no measurable alteration in the level of PRK3 associated with TPβ *(ii)*, PRK3 transiently dissociates from TPα *(i)* at 10 min post-agonist before re-associating following prolonged agonist stimulation (60 min). Agonist-stimulation of both TPα and TPβ leads to T-loop phosphorylation-dependent activation of PRK1, PRK2 and PRK3, where each of the kinases remain in the activated state for at least 60 min post-agonist. Thus, while PRK2 and PRK3, but not PRK1, transiently associate/disassociate from the given TPα/TPβ complex in response to agonist stimulation, the agonist-induced phosphorylation & activation of each of the PRKs is more sustained. Panel **B.** In PC-3 cells, TPα/TPβ is found associated in a complex with both PRK1 and PRK2. Agonist-activation of TPα/TPβ leads to activation of phosphatidylinositol 3′kinase (PI3′K) and, in turn, to activation of 3-phosphoinositide-dependent protein kinase-1 (PDK-1), the master-regulator AGC kinase that leads to subsequent activation of the PRKs through phosphorylation of the essential Thr within their activation loop (T-loop phosphorylation). Critically, in response to TPα/TPβ stimulation, activation of both PRK1 and PRK2 leads to chromatin remodelling (H3Thr11 phosphorylation) in prostate cells and to enhanced cell proliferation and migration, thereby exacerbating the pathological state in prostate cancer. Note; TPα/TPβ-regulation of G_q_/PLCβ, G_12_/Rho or of the ERK signalling cascades are not shown.

Based on our findings with the full-length PRKs, we propose a model (Figure 8A) whereby, under constitutive/basal conditions in the absence of agonist, PRK1 and/or PRK3 is recruited into a complex with both of the TPα and TPβ isoforms, while PRK2 distinctly forms a complex only with that of TPβ. In response to short-term U46619/TXA_2_-induced receptor activation (e.g at 10 min), while there is no measurable alteration in the level of PRK1 associated with either TPα or TPβ, a change occurs whereby PRK2 transiently dissociates from TPβ, and PRK3 transiently dissociates from TPα, coinciding with/facilitating the recruitment of PRK2 into a transient complex with TPα. Following prolonged agonist-activation of TPα/TPβ, PRK2 dissociates from TPα and is recruited once more into a complex with TPβ, while the association between TPβ and PRK3 is also restored. Furthermore, signalling through TPα/TPβ leads to T-loop phosphorylation and activation of PRK1 and PRK2 in PC-3 cells and this occurs in a mechanism-dependent on PI3′K/PDK1. Thus, while PRK2, but not PRK1, transiently associate/disassociate from the given TPα/TPβ complex in response to agonist stimulation, the agonist-induced T-loop phosphorylation & activation of each of these PRKs is more sustained. Critically, we also show that both PRK1 and PRK2, but not PRK3, are essential in mediating TXA_2_/U46619-induced chromatin remodelling (H3Thr11 phosphorylation) and neoplastic responses (cell proliferation, anchorage independent growth/colony formation, and cell migration) in prostate cancer cells. Hence, TP-mediated prostate tumour responses not only involves PRK1, but is also dependent on signalling through the related PRK2, involving a unique and dynamic series of complexes regulated in response to agonist-activation of the TPs.

Notably, following multiple attempts PRK3 did not form a complex with TPα or TPβ in PC-3 cells, despite showing associations in HEK293 cells that can be agonist-regulated and dependent on the C-terminal C2 and kinase domains of PRK3. Given the relatively lower expression of PRK3 in PC-3 cells, one possible explanation is that TPα/TPβ might preferentially associate with the more abundant PRK1 and PRK2 in this prostate cell line. However, even in the absence of PRK1 and PRK2 expression, following their specific disruption by *si*RNAs, PRK3 was not found to associate with TPα or TPβ in PC-3 cells (data not shown). Another possibility might be that there is a missing functional element in prostate cells, which would otherwise facilitate the association of TPα/TPβ with PRK3. Interestingly, a recent study has revealed that RhoC predominately associates with PRK3 in prostate cells leading to metastatic prostate tumour growth downstream of a hyperactive PI3′K pathway [44]. Hence, PRK3 may function as an effector of another branch of the PI3′K oncogenic signalling network in prostate cancer, which is potentially independent of the TXA_2_-TPα/TPβ signalling axis [43, 44]. While our data have provided evidence for the propensity of both TPα and TPβ to associate with and regulate PRK3, the biological significance of these findings remains to be clarified and will be the subject of future investigations. Specifically, it will be of interest to investigate the role of the TPα/TPβ-mediated PRK3 signalling in other neoplastic settings where both TXA_2_ and PRK3 are implicated.

As illustrated in the model in Figure 8B, TXA_2_/U46619-induced activation of the PRKs occurs in a mechanism dependent on signalling through PI3′K and subsequent activation of the master-regulatory AGC kinase PDK1, leading to T-loop phosphorylation and activation of PRK1/2 [37, 38]. These findings are entirely consistent with the established role for TPα/TPβ in the activation of both class 1A and class 1B PI3′K signalling cascades [27–29]. Furthermore, mutational analysis of the PRKs reveals that the stability of the complex formed between TPα/TPβ and the PRKs is dependent upon their T-loop phosphorylation which occurs in response to U46619-induced receptor activation. Therefore, it appears that through their ability to associate with TPα/TPβ, in response to receptor activation PRK1/2 undergoes enhanced PDK1-dependent phosphorylation at its T-loop, a phosphorylation event that is critical for regulating/stabilizing the association of TPα/TPβ with the PRKs.

Of crucial importance to the current study is that in response to receptor stimulation, activation of both PRK1 and PRK2 are involved in H3Thr11 phosphorylation, cell proliferation/migration in PC-3 cells, thereby providing evidence for the additional role of PRK2 in mediating TXA_2_-induced chromatin remodelling and malignant growth/metastasis in prostate cancer. From a mechanistic point of view, phosphorylation of H3Thr11 by PRK1 and/or by the related PRK2 accelerates the demethylation of histone 3 at lysine 9 (H3K9) by the de-methylases JMJD2C and lysine specific de-methylase (LSD) 1, thereby promoting AR-dependent gene expression and cellular growth and migration in prostate cancer [47, 53, 54]. In addition, it was recently established that PRK1-induced phosphorylated H3Thr11 serves as a binding platform for the WDR5 (WD-repeat containing protein 5) component of the WRAD complex, thereby leading to recruitment of the MLL1 methyltransferase complex, and consequent trimethylation of H3K4 and transcriptional activation of AR-target genes [55]. Our discovery, therefore, that signalling through TPα/TPβ can lead to PRK1/PRK2-induced H3Thr11 phosphorylation is truly exciting, and clearly highlights a potentially important role for TPα and/or TPβ in chromatin remodelling/epigenetic regulation in tumour progression. Furthermore, it is tempting to speculate that these TPα/TPβ-mediated effects may account, at least in part, for the clinically well-documented observation that during prostate cancer progression, AR-target gene expression is up-regulated even in the absence of testicular androgens or in the presence of AR antagonists, such as in the case of CRPC. It will therefore be of significant interest to now investigate whether TPα/TPβ- mediated PRK1/2 activation can lead to further chromatin modifications in prostate cells, including recruitment of the MLL1 complex and subsequent trimethylation of H3K4, thereby leading to an altered gene expression programme in prostate tumour cells. The importance of the PRK family members in transcriptional regulation is further endorsed by recent reports that key members of class IIa histone deacetylases are phosphorylated by PRK1/2. More specifically, HDAC−5, −7 and −9 but not −4 are phosphorylated by PRK1/2 within their nuclear localisation signal, thereby impairing histone deacetylase nuclear transport and promotion of transcriptional activation [56].

Interestingly, through interactions involving its N-terminal domain, PRK2 is known to form homodimers, also raising the possibility of the existence of heterodimers between members of the structurally related PRK family [57]. Hence, given our discovery that TXA_2_-induced H3Thr11 phosphorylation, as well as cell proliferation/migration appear to be almost equivalently dependent on signalling through both PRK1 and PRK2, despite differences in their relative expression levels and modes of regulation in PC-3 cells, we sought to explore the possibility that the ability of PRK2 to associate with TPα/TPβ might occur in a mechanism which is dependent on the related PRK1, or *vice versa*. In preliminary experiments, we found that endogenous expression of PRK1 may be a pre-requisite for the association of PRK2 with TPα/TPβ which occurs in PC-3 cells. Hence, although clarification of our observation requires detailed investigation, this raises the possibility of the existence of a potential TPα/TPβ signalling complex in prostate tumour cells, comprising the receptor and both PRK1 and PRK2, where PRK1 may function to facilitate binding of PRK2 to TPα/TPβ and/or PRK1. However, the fact that PRK1 is constitutively bound to both TPα and TPβ, but that PRK2 does not bind to TPα but binds to TPβ under those same conditions, in the absence of agonist, suggests that PRK1 is not simply serving as a binding platform for PRK2 binding. Hence, additional studies are necessary to understand the micro-organisation of TP:PRK protein-protein complexes generated and, in turn, may facilitate the design of drugs to block critical protein interactions in cases where the protein associations leads to disease [58]. Therefore, given the evidence presented herein for the critical role of the TPα/TPβ-PRK1/PRK2 signalling axis in prostate tumorigenesis, it will be of interest to determine the precise molecular architecture of the protein complexes which exist between the TPs and PRK1/PRK2, including identification of the region(s) of TPα/TPβ which mediate their association with PRK2, and determining whether PRK1 can directly associate with PRK2. Furthermore, it will also be crucial to establish if the TPα/TPβ-PRK1/PRK2 signalling axis plays a role in tumour progression in other carcinomas where both the TP and the PRKs are implicated including in breast and bladder cancer [42, 59].

In conclusion, as outlined in Figure 8B, our study has uncovered a novel signalling pathway involving TPα/TPβ and both PRK1 and PRK2, providing a mechanism for how agonist activation of the TP may exacerbate prostate cancer, including CRPC. However, intriguingly while both PRK1 and PRK2 associate with TPα and TPβ, they do so in a radically different manner under both basal and agonist-regulated conditions, including where both TP isoforms show different associations with PRK2. Bearing in mind the crucial role for PRK1 and PRK2 in the epigenetic regulation of events associated with prostate tumour progression/metastasis [47, 55], our discovery of the propensity of TPα/TPβ to induce PRK1/PRK2-mediated chromatin remodelling and neoplastic responses in prostate cells is highly significant, and highlights the potential for TPα and/or TPβ as a therapeutic target in the treatment of prostate cancer including CRPC and potentially in other cancers in which aberrant TP:PRK1 and/or PRK2 signalling is implicated. Furthermore, our study provides a mechanistic explanation, at least in part, for the prophylactic benefits of Aspirin in reducing certain cancer risks by lowering overall TXA_2_ levels.

## MATERIALS AND METHODS

### Materials

The pCMVTag2b vectors and the QuikChange site-directed mutagenesis (SDM) kit were obtained from Agilent. U46619 was obtained from Cayman Chemical Company. Goat polyclonal anti-PRK1 (sc-1842), goat polyclonal anti-PRK2 (sc-6979), mouse polyclonal anti-PRK2 (sc-271971), goat polyclonal anti-histone H3 (sc-8654), normal rabbit immunoglobulin G (IgG; sc-2027), HRP-conjugated goat anti-rabbit secondary antibody (sc-2004), HRP-conjugated goat anti-mouse secondary antibody (sc-2005) and HRP-conjugated mouse anti-goat secondary antibody (sc-2354) were obtained from Santa Cruz Biotechnology. Mouse monoclonal anti-HA 101R (clone 16B12) antibody was from Covance. Mouse monoclonal anti-FLAG-HRP-conjugated antibody, LY294002, GSK2334470, PKC412 (Midostaurin), colcemid, thiazolyl blue tetrazolium bromide (MTT), Protein A-Sepharose and Protein G-Sepharose were obtained from Sigma. Mouse monoclonal anti-HDJ2 (DNAJ) antibody and Dharmafect 2 transfection reagent were from ThermoFisher Scientific. Rabbit polyclonal anti-phospho-H3Thr11 (39151) was obtained from Active Motif. Rabbit polyclonal anti-PRK3 (NBP1-30102) antibody was obtained from Novus Biologicals. Rat monoclonal anti-HA-HRP-conjugated antibody (3F10) was from Roche Applied Science. Rabbit polyclonal anti-phospho-PRK1^Thr774^/PRK2^Thr816^ (#2611) was obtained from Cell Signalling Technology. Boyden chamber cell culture inserts (#353097) were obtained from BD Biosciences. Effectene transfection reagent was from Qiagen. H-89 dihydrochloride and Gö6983 were obtained from Merck Millipore. All oligonucleotides were synthesized by Genosys Biotechnologies and all small interfering RNAs (*si*RNA) were from Eurofins Scientific.

### Expression plasmids

The plasmids pCMVTag2b:PRK1, pCMVTag2b:PRK1^1–357^, pCMVTag2b:PRK1^1–594^, pCMVTag2b:PRK1^561–942^, and pCMVTag2b:PRK1^K644E^ have been previously described [35]. The plasmid pCMVTag2b:PRK2 was generated by sub-cloning the full-length, PCR-amplified PRK2 sequence from pEGFP-C1:PRK2 [60], obtained from Dr Peter Parker (Protein Phosphorylation Laboratory, London Research Institute, London, UK), in-frame with the translation of the FLAG epitope tag. The plasmid pCMVTag2b:PRK3 was generated by sub-cloning the full-length, PCR-amplified PRK3 sequence from pOTB7:PRK3, obtained from Genome Cube (IRAUp969B07105D), in-frame with the translation of the FLAG epitope tag. The plasmids pCMVTag2b:PRK1^T774A^, pCMVTag2b:PRK2^1–362^, pCMVTag2b:PRK2^1–621^, pCMVTag2b:PRK2^577–984^, pCMVTag2b:PRK2^K686E^, pCMVTag2b:PRK2^T816A^, pCMVTag2b:PRK3^1–309^, pCMVTag2b:PRK3^1–539^, pCMVTag2b:PRK3^512–889^, pCMVTag2b:PRK3^K588E^ and pCMVTag2b:PRK3^T718A^ were generated by either sub-cloning of the respective PCR-amplified subfragments into pCMVTag2b such that they were in-frame with the N-terminal FLAG epitope tag or by QuikChange SDM using the respective full-length PRK-containing plasmid as template. In all cases, sequences of the specific primers used are listed in Supplemental Table 1. All plasmids were validated by DNA sequence analysis.

### Cell culture and transfections

Human embryonic kidney (HEK).TPα, HEK.TPβ, and HEK.β-Gal cells, stably overexpressing haemagglutinin (HA)-tagged forms of TPα, TPβ, and β-galactosidase (β-Gal), respectively, have been previously described [35] and were grown in minimal essential medium (MEM), 2 mM L-glutamine and 10% (v/v) fetal bovine serum (FBS). Routinely, approx. 48 hr prior to transfection, cells were plated at a density of 2 × 10^6^ cells/10 cm dish in growth medium (8 ml). Thereafter, cells were transiently transfected with 400 ng of pADVA and 1.6 μg of pCMV-based vectors using Effectene and routinely harvested 48–72 hr post-transfection. The prostate carcinoma PC-3 cells were obtained from the American Type Culture Collection (ATCC) and were cultured in RPMI 1640, 2 mM L-glutamine, and 10% FBS (complete medium). For serum-free media conditions, cells were cultured in their respective growth medium in the complete absence of FBS (0%) for times specified for the given study. All cells were grown at 37°C in a humid environment with 5% CO_2_ and were confirmed mycoplasma free.

### Immunoprecipitations

HEK.TPα, HEK.TPβ and HEK.β-Gal cells were transfected and cultured as described above. Alternatively, for immunoprecipitation of endogenous TP/PRK complexes, PC-3 cells were seeded on 10 cm dishes and cultured at 37°C, typically for 72 hr or until 85% confluent. In all cases, prior to immunoprecipitation, cells were washed in serum-free media and either incubated with vehicle (0.01% EtOH) or U46619 (1 μM) in serum free media for 0–60 min, or as indicated in the figure legends. Thereafter, cells were washed twice in ice-cold PBS and incubated in Radioimmune Precipitation (RIP) buffer (20 mM Tris-Cl, pH 8.0, 150 mM NaCl, 10 mM EDTA, 1% (v/v) NP-40, 1% (w/v) sodium deoxycholate, 0.1% (w/v) SDS, 1 mM sodium orthovanadate, 1 mM PMSF, 4 μg/ml leupeptin, 2.5 μg/ml aprotinin; 800 μl/10 cm dish) on ice for 10 min to lyse the cells. Lysates were homogenised by passing through needles of decreasing gauge (21–26) and clarified by centrifugation at 13, 000 *g* for 5 min to pellet the cell debris. The supernatant (containing the soluble lysate) was removed and added to a fresh micro-centrifuge tube and an aliquot (typically 50 μl/approx. 50 μg) was retained for analysis of protein expression in whole cell lysates. The remaining lysate (approx. 750 μl) was used for immunoprecipitation, using the specified antibody, typically anti-HA 101R (2 μl) for HEK.TPα, HEK.TPβ and HEK.β-Gal or affinity purified anti-TPα (6 μg) and anti-TPβ (6 μg) specific antibodies for PC-3 cells or, as controls, using the equivalent amount of the pre-immune IgG to pull-down the relevant protein through overnight incubation at 4°C on a rotator. Thereafter, the lysates were incubated for 1 hr with Protein G-Sepharose (50% slurry in RIP buffer; 15 μl) or Protein A-Sepharose (50% slurry in RIP buffer; 30 μl), prior to washing with at least four changes of RIP buffer followed by four changes of PBS. Immunoprecipitates were then subjected to immunoblotting versus either anti-FLAG-HRP, anti-PRK1, anti-PRK2, anti-PRK3 or anti-HA-HRP antibodies.

### Determination of PRK activation levels

HEK.TPα and HEK.TPβ cells were transfected and cultured as described above. Alternatively, PC-3 cells were seeded on 10 cm dishes and cultured at 37°C, typically for 72 hr or until 85% confluent. In all cases, some 16 hr prior to beginning experiments, the culture media was removed and cells were washed with serum-free media followed by incubation in serum-free media (16 hr). Cells were then washed twice in ice-cold PBS, harvested by scraping and suspended in PBS supplemented with protease inhibitors (10 mM Sodium Fluoride, 1 mM Sodium orthovanadate, 4 μg/ml leupeptin, 2.5 μg/ml aprotinin, 0.5 mM PMSF, 1 mM Benzamidine-HCl). Protein concentrations were determined using the Bradford assay and cells were divided into aliquots (100 μg/aliquot). Aliquots were then centrifuged at 2, 000 g for 2 min at 4°C and re-suspended in HBSS-HB (Hank's Balanced Salt Solution; supplemented with 10 mM HEPES, 0.1% (w/v) BSA and protease inhibitors). Cell suspensions were then equilibrated for 30 min at 37°C prior to incubation with either vehicle (0.00005–0.01% EtOH) or U46619 (1 μM, or in concentration-response studies at 0–1 μM) for 0–60 min. Alternatively, where the effects of kinase inhibition or TP antagonism were investigated, equilibrated cell suspensions were first incubated with either 10 μM LY294002, 0.1 μM GSK2334470, 10 μM H-89, 1 μM Go6983, 50 nM PKC412, 1 μM SQ29, 548 or with the respective drug vehicle (0.01% DMSO or 0.01% EtOH) for 30 min prior to incubation with U46619. Following stimulations, cells were harvested by centrifugation (4000 g, 2 min, 4°C) and re-suspended in ice-cold PBS (50 μl) supplemented with protease inhibitors. SDS-PAGE sample buffer (2X; 50 μl) was added. Samples were then homogenised, denatured by boiling (1 min) followed by centrifugation at 13, 000 g (1 min) and incubation at 37°C for 15 min. Finally, samples were resolved on SDS-PAGE gels and immunoblotted versus anti-phospho-PRK1^Thr774^/PRK2^Thr816^ or anti-HDJ2, followed by chemiluminescence detection.

### Disruption of PRK1/2/3 expression by small interfering (si)RNA

For *si*RNA experiments, PC-3 cells were routinely plated at a density of 2 × 10^5^ cells/35 mm well in complete medium (2 ml). Thereafter, PC-3 cells were transiently transfected with 30 nM *si*RNA specifically targeting either PRK1, PRK2, or PRK3 and, as a control, with a nonsense scrambled control *si*RNA oligonucleotide (5′-AATTCTCCGAACGTGTCACGT-3′), using Dharmafect 2 transfection reagent (4 μl per 35 mm well). For experiments involving *si*RNA-mediated disruption of PRK1, 2 and 3, all data was validated using at least two independent *si*RNAs where the following specific *si*RNA sequences were used: PRK1–1 (5′-CCUCGAAGAUUUCAAGUUC-3′), PRK1–2 (5′-GCACUGUGCUUAAGCUGGA-3′), PRK2–1 (5′-GCACCCAUUUUUCCGGCUA-3′), PRK2–2 (5′-GCACCCAUUUUUCCGGCUA-3′), PRK3–1 (5′-GGAAAUACUACGCCAUCAA-3′) or PRK3–2 (5′-AGACCUUUGUCAUCCCACU-3′). Cells were routinely harvested at 3, 4 or 6 day post-transfection, and knockdown of PRK1, PRK2 or PRK3 expression was confirmed by immunoblotting using anti-PRK1, anti-PRK2, or anti-PRK3 specific antibodies, respectively. Membranes were also screened using anti-HDJ2 antibody to confirm uniform protein loading. For proliferation assays, cells were harvested 48 hr post-transfection and re-seeded onto 96 well plates in complete growth medium (2000 cells/well; 150 μl) as described below. For colony formation assays, cells were harvested some 48 hr post-transfection and re-seeded into soft agar (2 × 10^4^ cells/35 mm well), as outlined below. For transwell migration assays, some 48 hr post-transfection, cells were routinely placed in serum-free media for 24 hr before experiments were performed, as described below.

### Investigation of histone H3 Thr11 (H3Thr11) phosphorylation

To examine the effect of U46619 on H3Thr11 phosphorylation, PC-3 cells were plated at 1 × 10^6^ cells/10 cm dish in complete culture medium (8 ml) and grown at 37°C for 48 hr, such that the cells reached approximately 85% confluency. Cells were stimulated with U46619 (50 nM, or in concentration-response studies, 0–1 μM; 0–240 min or 24 hr) or, as controls, with colcemid (50 ng/ml; 24 hr) or drug vehicle (0.0005% EtOH; 0–240 min or 24 hr). The cells were then harvested by sequential extraction to remove soluble cytoplasmic and nucleoplasmic proteins to obtain nuclear extracts (containing histones) as described previously [61]. In brief, cells were washed with ice-cold PBS and incubated in hypotonic buffer (10 mM Tris-Cl, pH 8.0 and 1 mM KCL, 1.5 mM MgCl_2_, 1 mM PMSF, 50 μM leupeptin and 1 mM DTT; 1 ml) for 30 min at 4°C with rotation to lyse the cells. The lysate and nuclei were separated by centrifugation (10, 000 *g*) and the nuclei were re-suspended in H_2_SO_4_ (0.4 N; 400 μl) and incubated overnight at 4°C with rotation. Nuclear debris was removed by centrifugation (16, 000 *g*) and the histone-containing supernatant was transferred to a fresh microfuge tube. Histones were then precipitated by adding trichloroacetic acid (100% w/v; 132 μl) drop-wise to the histone solution. Pellets were then washed twice with ice-cold acetone (500 μl) to remove the acid, air-dried and re-suspended in double-distilled H_2_O (100 μl). Thereafter, histone concentrations were determined using the Bradford assay. For immunoblot analysis, nuclear extracts (2.5 μg) were resolved on 15% SDS-page gels and transferred onto PVDF membranes. Thereafter, membranes were screened using anti-Phospho-H3Thr11 antibody or anti-Histone H3 to confirm uniform loading.

### Transwell migration assays

PC-3 cells were seeded on 10 cm dishes in complete culture medium (8 ml) and allowed to grow at 37°C until the cells reached approximately 80% confluency. Thereafter, the media was replaced with serum-free medium for at least 16 hr before performing transwell assays, using Boyden Chambers (Falcon #353097, 8.0 μM pore size, 24-well plate format). Boyden Chambers were coated overnight with fibronectin (5 μg/ml), prior to beginning assays. For migration assays, 1 × 10^5^ cells, in serum-free culture media (500 μl), were placed in the top chamber. After the cells settled (approx. 30 min), culture medium (500 μl; 10% FBS) was added to the bottom chamber and cells were incubated with U46619 (10 nM or, in concentration response studies, 5–250 nM) or vehicle (0.0001% EtOH) in duplicate wells. Following incubation for 8 hr, the cells remaining in the top chamber were removed by cotton swabs, and the migrated cells were fixed in PFA (3.7%), stained with crystal violet dye (0.25%), washed in H_2_O and allowed to air-dry. The cells were then viewed and photographed using a light microscope at 20 × magnification. For analysis, the numbers of migrated cells from at least three independent fields of view were counted.

### Colony formation assays

PC-3 cells were seeded at 2 × 10^4^ cells/35 mm well in 2 ml of 0.35% agar prepared in RPMI with 2x supplements (20% FBS; 4 mM L-glutamine). The 0.35% soft agar layer was anchored with 2 ml of 0.5% agar previously prepared in RPMI with 2x supplements. Thereafter, the cells were allowed to settle in the soft agar layer for approx. 1 hr before adding a further 2 ml of complete culture medium (RPMI, 10% FBS, 2 mM L-Glut) to the wells. Cells were treated with U46619 (10 nM or, in concentration response studies, 5–250 nM) or vehicle (0.0001% EtOH). Media and treatments were refreshed every 72 hr for the duration of the experiment. Some 10 days post-seeding, cells were fixed in paraformaldehyde (3.7%), stained with crystal violet dye (0.05%), washed in H_2_O and allowed to air-dry. The number of colonies formed in triplicate wells was analysed, where colonies were manually counted using a light microscope (20X magnification) from 10 independent fields of view.

### Proliferation assays

PC-3 cells (2000 cells/well) were seeded in complete culture medium (RPMI, 10% FBS, 2 mM L-Glut; 150 μl) in 96-well plates and grown for 24 hr at 37°C. Thereafter, the media was replaced with serum-free media containing U46619 (10 nM or, in concentration response studies, 5–250 nM) or vehicle (0.0001% EtOH), with cells then cultured for an additional 48 hr. Thereafter, MTT reagent (5 mg/ml; 50 μl) was added to each well and the cells were further incubated at 37°C for 3 hr. The MTT/media mix was then removed and replaced with DMSO (200 μl/well) to elute the purple formazan crystals. Levels of formazan were measured by reading the absorbance at 540 nm from triplicate wells.

### Data analyses

Statistical analyses of differences were carried out using the unpaired Student's *t* test, or one-way ANOVA followed by post hoc Dunnett's multiple comparison *t* test employing GraphPad Prism throughout. *P* values of ≤ 0.05 were considered to indicate a statistically significant difference. As relevant, single, double and triple asterisk symbols signify *p* ≤ 0.05. ≤0.01 and ≤0.001, respectively. Semi-quantitative densitometry was performed on appropriately exposed immunoblots, wherein the Gel Analysis Tool of the ImageJ image analysis package (v1.45s) was used to obtain the absolute intensity for each experimental band and the corresponding control band.

## SUPPLEMENTARY FIGURES AND TABLE



## Abbreviations

AA: arachidonic acid
ADT: androgen deprivation therapy
AR: androgen receptor, CRPC, castrate resistant prostate cancer
COX: cyclooxygenase/prostaglandin G2/H2 synthase
C-tail: carboxyl-terminal tail
DHT: dihydrotestosterone
ERK: extracellular signal regulated protein kinase
FBS: foetal bovine serum
GPCR: G protein-coupled receptor
HA: hemagglutinin
HEK: human embryonic kidney
H3Thr11: histone H3 threonine 11
PCa: prostate cancer
PDK-1: 3-phosphoinositide-dependent protein kinase-1
PG: prostaglandin
PI3′K: phosphatidylinositol 3′kinase
PK: protein kinase
PRK: Protein kinase C-related kinase
RBD: Rho binding domain
siRNA: small interfering RNA
TP: T Prostanoid receptor
TXA_2_: thromboxane (TX) A_2_
