# Supplementary material for: Protein kinase C-related kinase 1 and 2 play an essential role in thromboxane-mediated neoplastic responses in prostate cancer
Source: Oncotarget. 2015 Jul 20;6(28):26437–56. doi: 10.18632/oncotarget.4664 (PMC4694913; doi:10.18632/oncotarget.4664)
Supplement: Supplementary file 1 [file oncotarget-06-26437-s001.pdf]

SUPPLEMENTARY FIGURES AND TABLE

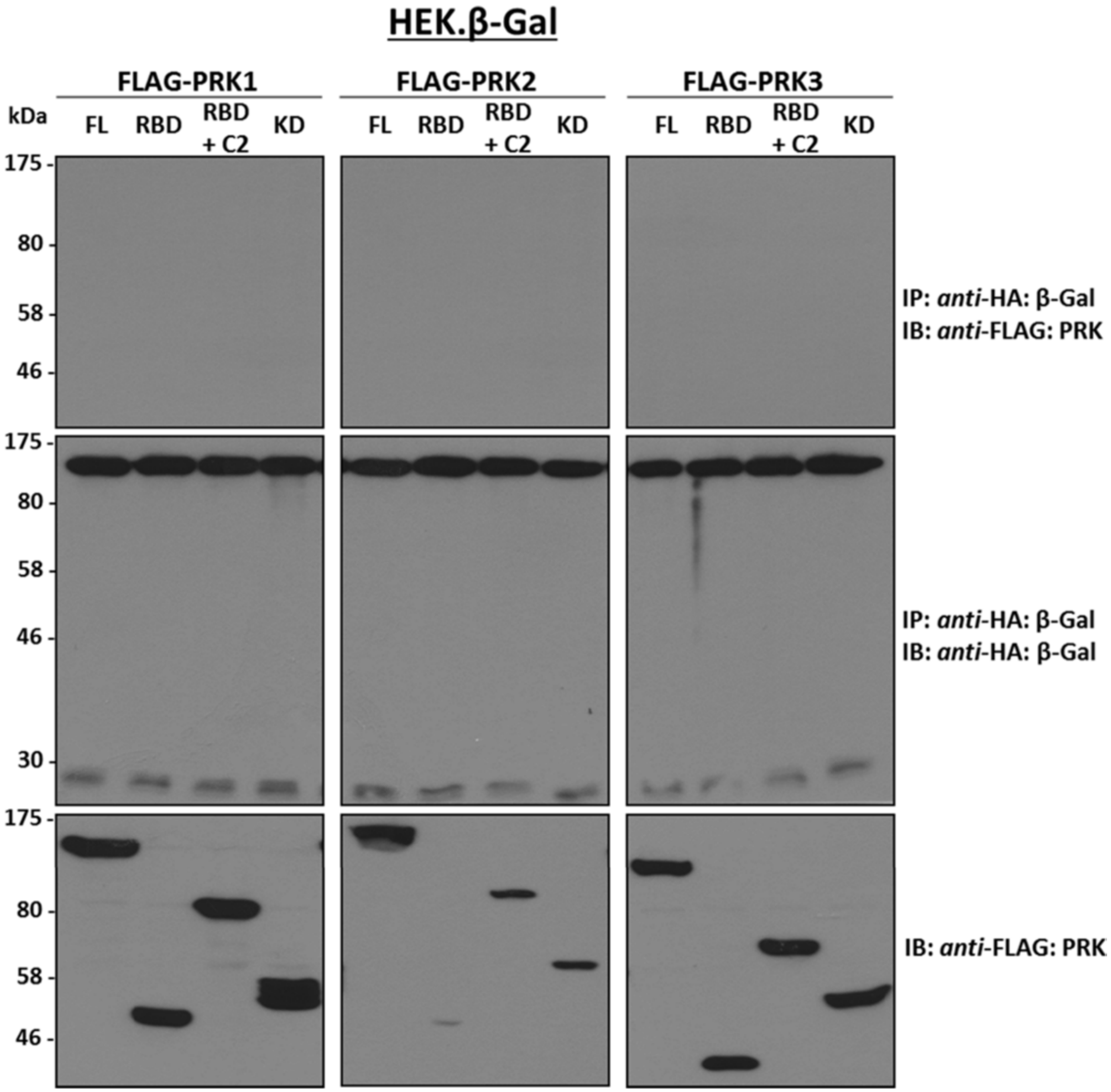

Supplementary Figure S1: Association of PRK1, PRK2 and PRK3 with TPα and TPβ in HEK 293 cells. As controls for the experiments in Figure 2D & 2E, HEK 293 cells stably over-expressing HA-tagged β-galactosidase (β-gal) and co-transfected with FLAG-tagged PRK1, PRK2 and PRK3 (FL, RBD, RBD+C2, kinase domain/KD) were immunoprecipitated with *anti*-HA antiserum and then immunoblotted (IB) with *anti*-FLAG or *anti*-HA (upper and middle panels, respectively). To verify uniform expression of the PRKs, aliquots of the whole cell lysates (20 µg/lane) were IB with *anti*-FLAG antiserum (lower panels). Data $n \geq 3$.

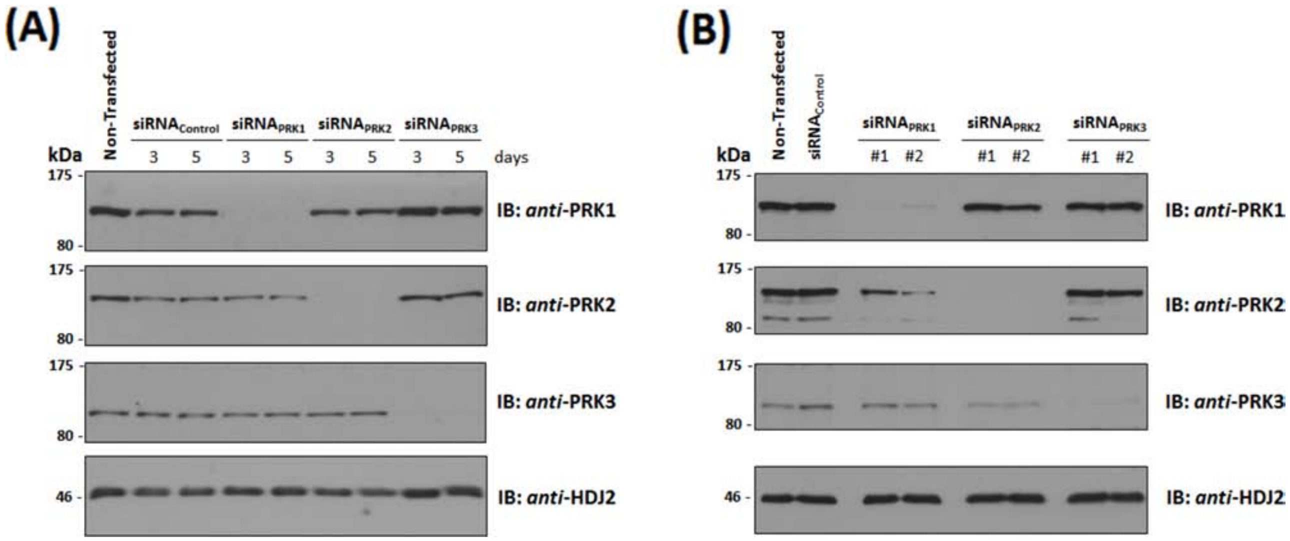

Supplementary Figure S2: Validation and specificity of siRNAs to PRK1, PRK2 and PRK3. Panel **A.** PC-3 cells were transfected with 30 nM *si*RNA$_{PRK1}$, *si*RNA$_{PRK2}$, *si*RNA$_{PRK3}$ or, as a control, with a nonsense scrambled *si*RNA (*si*RNA$_{control}$) where non-transfected cells served as a reference. Aliquots of whole cell lysates (20 µg/lane) were analysed 3 day and 5 day post-transfection by immunoblotting with *anti*-PRK1, *anti*-PRK2, *anti*-PRK3 antisera and back-blotting with *anti*-HDJ2 antiserum, to verify uniform protein loading, respectively. Data shown; $n \geq 3$. Panel **B.** PC-3 cells were transfected with 30 nM of two independent siRNAs (#1/#2) directed to each of PRK1, PRK2, PRK3 or, as a control, with a nonsense scrambled *si*RNA (*si*RNA$_{control}$) where non-transfected cells served as a reference. Aliquots of whole cell lysates (20 µg/lane) were analysed 5 days post-transfection by immunoblotting with *anti*-PRK1, *anti*-PRK2, *anti*-PRK3 antisera and back-blotting with *anti*-HDJ2 antiserum, to verify uniform protein loading, respectively. Data shown; $n \geq 3$.

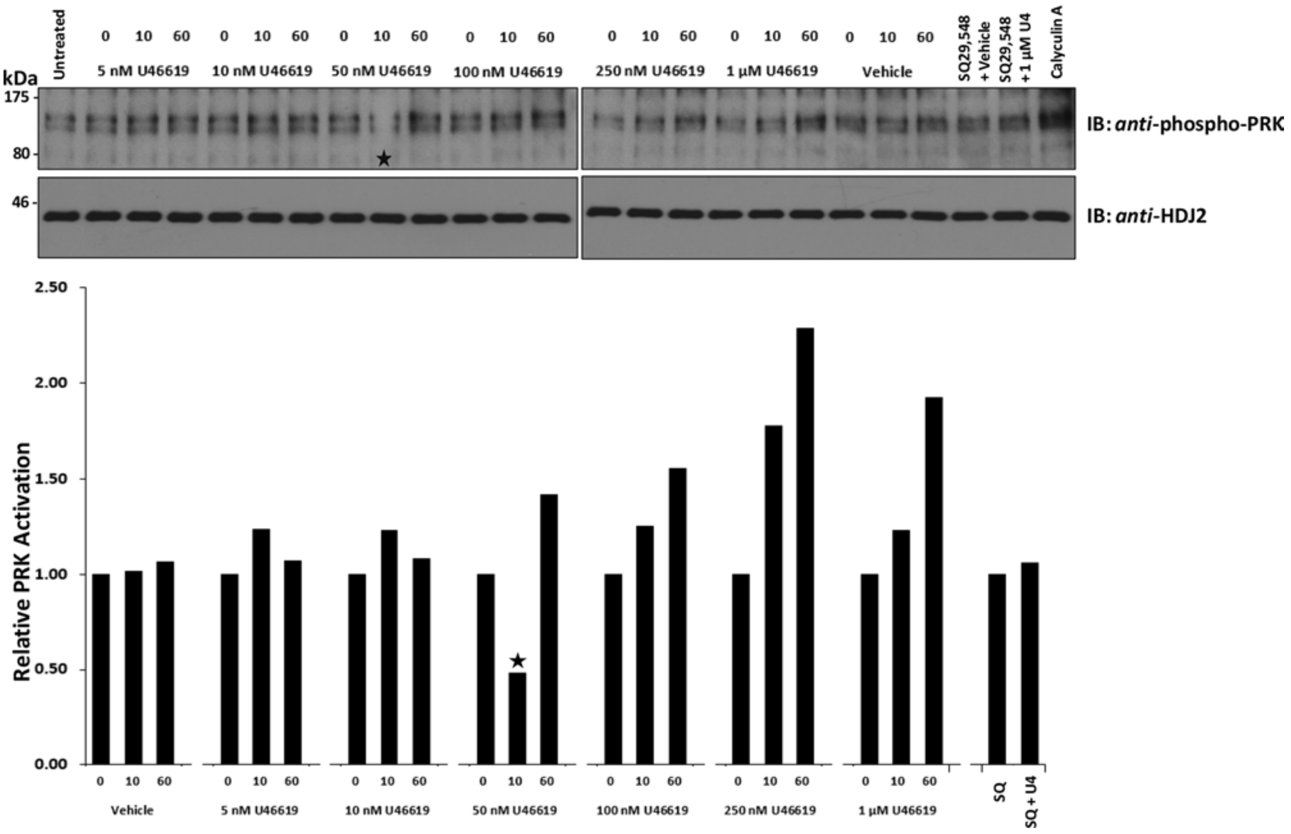

Supplementary Figure S3: Concentration-dependent effect of U46619 on PRK1 and PRK2 activation in PC-3 cells. PC-3 cells were incubated with U46619 (0, 5, 10, 50, 100, 250, 1000 nM) or with the drug vehicle (0–0.01% EtOH) for 0, 10 and 60 min. Aliquots of the whole cell lysates (20 µg/lane) were immunoblotted (IB) with *anti*-phospho-PRK1^{Thr774}/PRK2^{Thr816}/PRK3^{Thr718} to detect PRK activation/T-loop phosphorylation (upper panels) and then back-blotted with *anti*-HDJ2 antisera (lower panels), to verify uniform protein loading in each of the lanes. The bar charts show the mean overall levels of PRK activation (phospho-PRK1^{Thr774}/PRK2^{Thr816}) relative to HDJ-2 levels, as determined by densitometry, where levels in the vehicle-treated cells are assigned *a* value of 1. Note, in the representative data shown, the asterisks (*) depicts that there was a transfer problem with the data for 50 nM U46619 at 10 min, resulting in decreased densitometric detection. However, the overall data is consistent with a U46619-dependent increase in PRK1/PRK2 activation where the maximal response was found at 250 nM U46619 following 60 min stimulation.

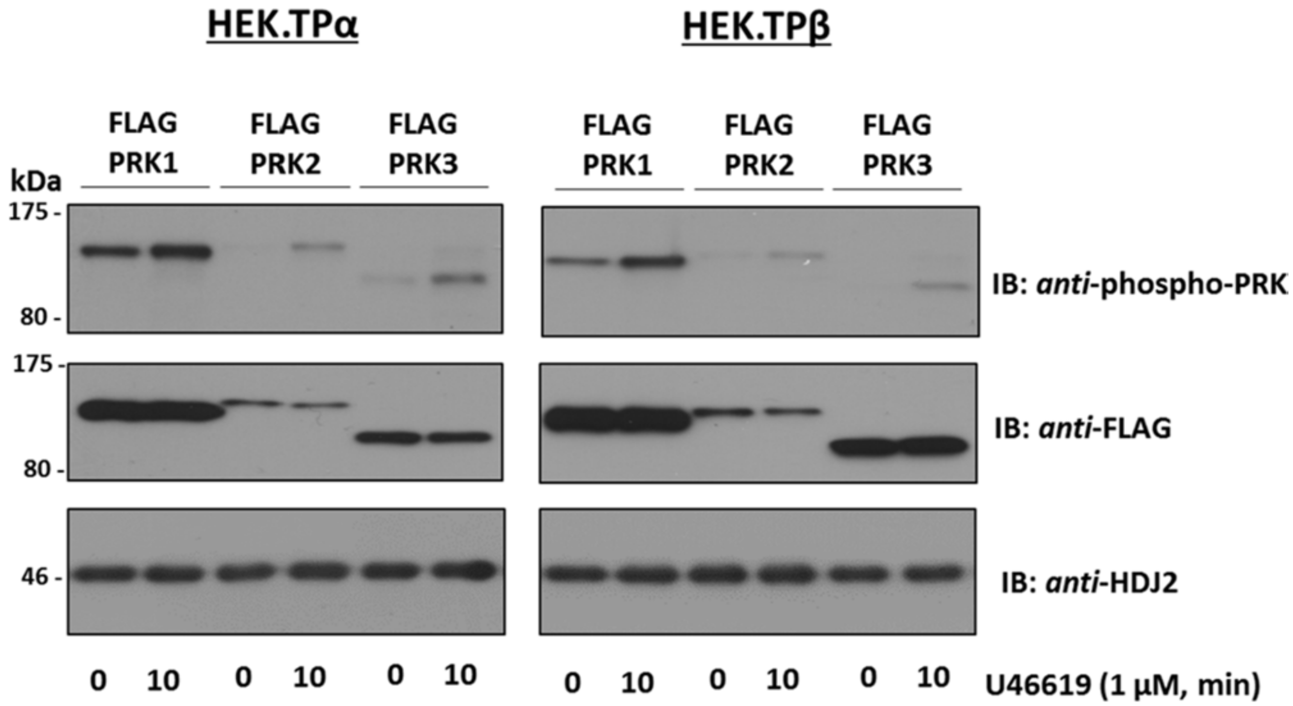

Supplementary Figure S4: Specificity of the Anti-Phospho T-loop Activation Antibody. HEK.TPα and HEK.TPβ cells, transiently transfected with FLAG-tagged PRK1, PRK2 or PRK3 were incubated with U46619 (1 µM; 10 min) or with vehicle (0.01% EtOH). Aliquots of the whole cell lysates (20 µg/lane) were immunoblotted (IB) with *anti*-phospho-PRK1^{Thr774}/PRK2^{Thr816}/PRK3^{Thr718} to detect T-loop phosphorylation (upper panels) and then successively back-blotted with *anti*-FLAG and *anti*-HDJ2 antisera (middle and lower panels), to verify uniform expression of the PRKs and protein loading, respectively. Data shown; $n \geq 3$.

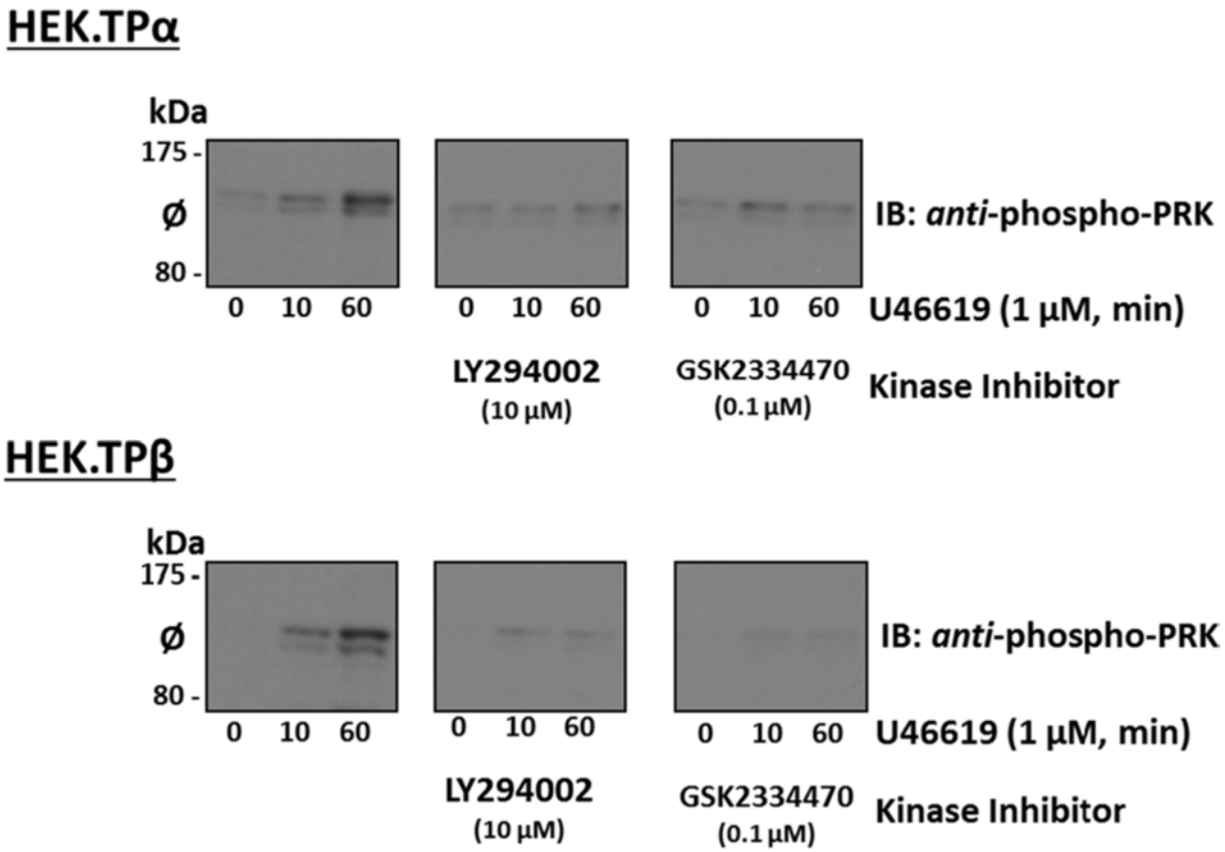

Supplementary Figure S5: Influence of TP Agonist on the Activation of PRK1, PRK2 and PRK3. HEK.TPα or HEK.TPβ cells, serum-starved (0% FBS, 16 hr), were pre-incubated for 30 min with the listed protein kinase inhibitors or, as controls, with drug vehicle (0.001% DMSO) prior to stimulation with U46619 (1 µM) or vehicle (0.01% EtOH) for 0, 10 or 60 min. Cells were harvested and aliquots (20 µg/lane) were immunoblotted (IB) with *anti*-phospho-PRK1^{Thr774}/PRK2^{Thr816}/ PRK3^{Thr718} (T-loop phosphorylation) and thereafter with *anti*-HDJ2 antisera to verify equal protein loading. Data $n \geq 3$.

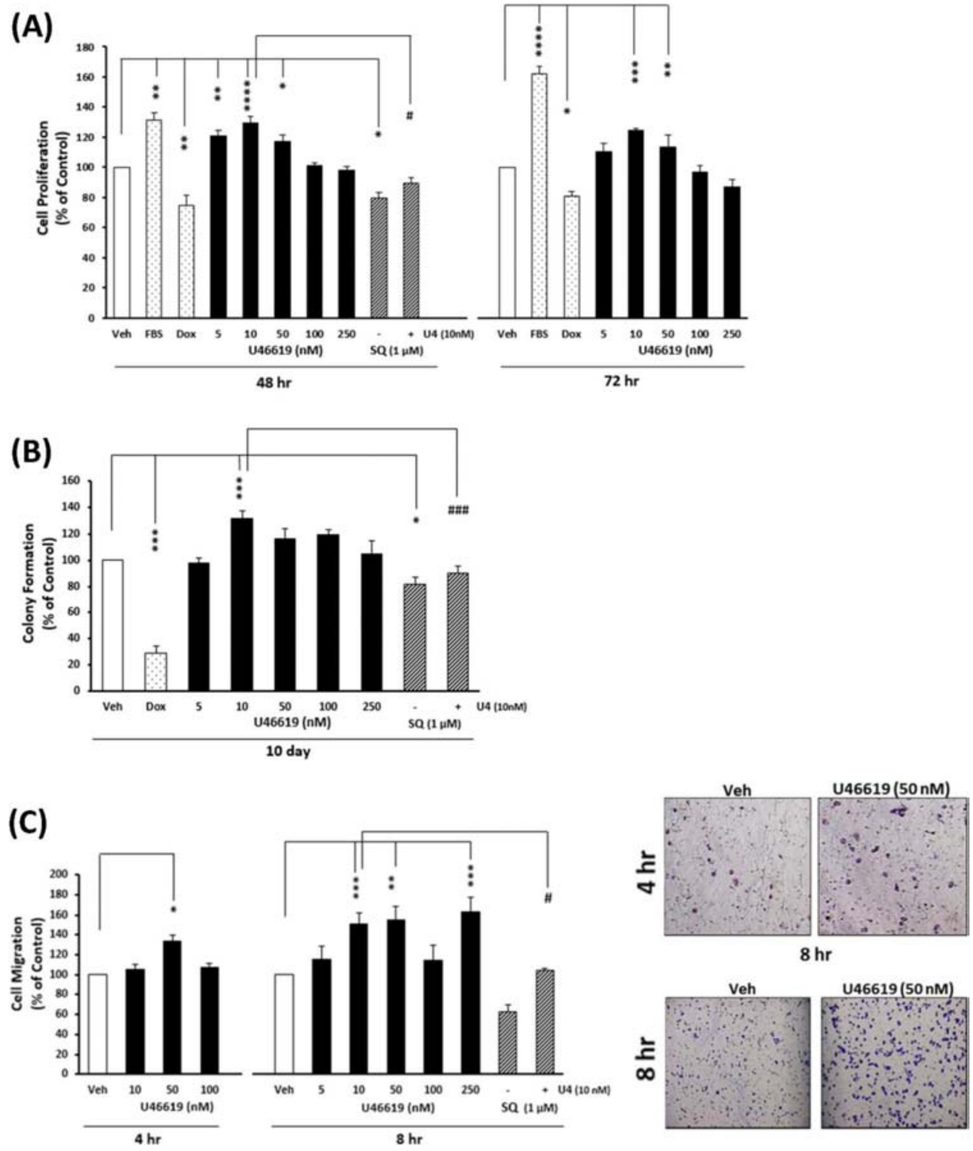

Supplementary Figure S6: Effect of TP agonist stimulation on proliferation, colony formation and migration of PC-3 cells. Panel **A.** For analysis of cell proliferation, serum starved PC-3 cells were incubated with U46619 (U4, 5 – 250 nM; black bars), SQ29548 (1 μM), U46619 (10 nM) plus SQ29548 (1 μM) or, as controls, vehicle (0.0001% EtOH), FBS (10%), Docetaxel (Dox; 20 nM) for either 48 or 72 hr, as indicated. Panel **B.** For analysis of colony formation in soft agar, serum starved PC-3 cells were incubated with U46619 (U4, 5 – 250 nM; black bars), SQ29548 (1 μM), U46619 (10 nM) plus SQ29548 (1 μM) or, as controls, Docetaxel (Dox; 20 nM), or vehicle (0.0001% EtOH) and colony formation assessed 10 days after drug treatment. Panel **C.** For analysis of migration, serum starved PC-3 cells were incubated for 4 or 8 hr with U46619 (U4, 5 – 250 nM; black bars), SQ29548 (1 μM), U46619 (10 nM) plus SQ29548 (1 μM) or vehicle (0.0001% EtOH). In *Panels A-C*, the bar charts show mean relative levels of PC-3 cell proliferation, colony formation and migration (± SEM, $n \geq 3$), respectively, where levels in the vehicle-treated cells are assigned *a* value of 100%. The asterisks signify that levels of proliferation, colony formation or migration by PC-3 cells were significantly increased in response to U46619 or FBS or significantly decreased in response to SQ29, 548 or Docetaxel compared with vehicle treated cells; # indicates that levels of U46619- induced responses by PC-3 cells were significantly decreased in the presence of SQ29, 548. In these cases, single, double, triple and quadruple symbols signify $p < 0.05$, 0.01, 0.001, 0.0001 respectively. Along with the bar charts, representative images of migrated PC-3 cells are shown, as viewed and photographed using a light microscope at 20X.

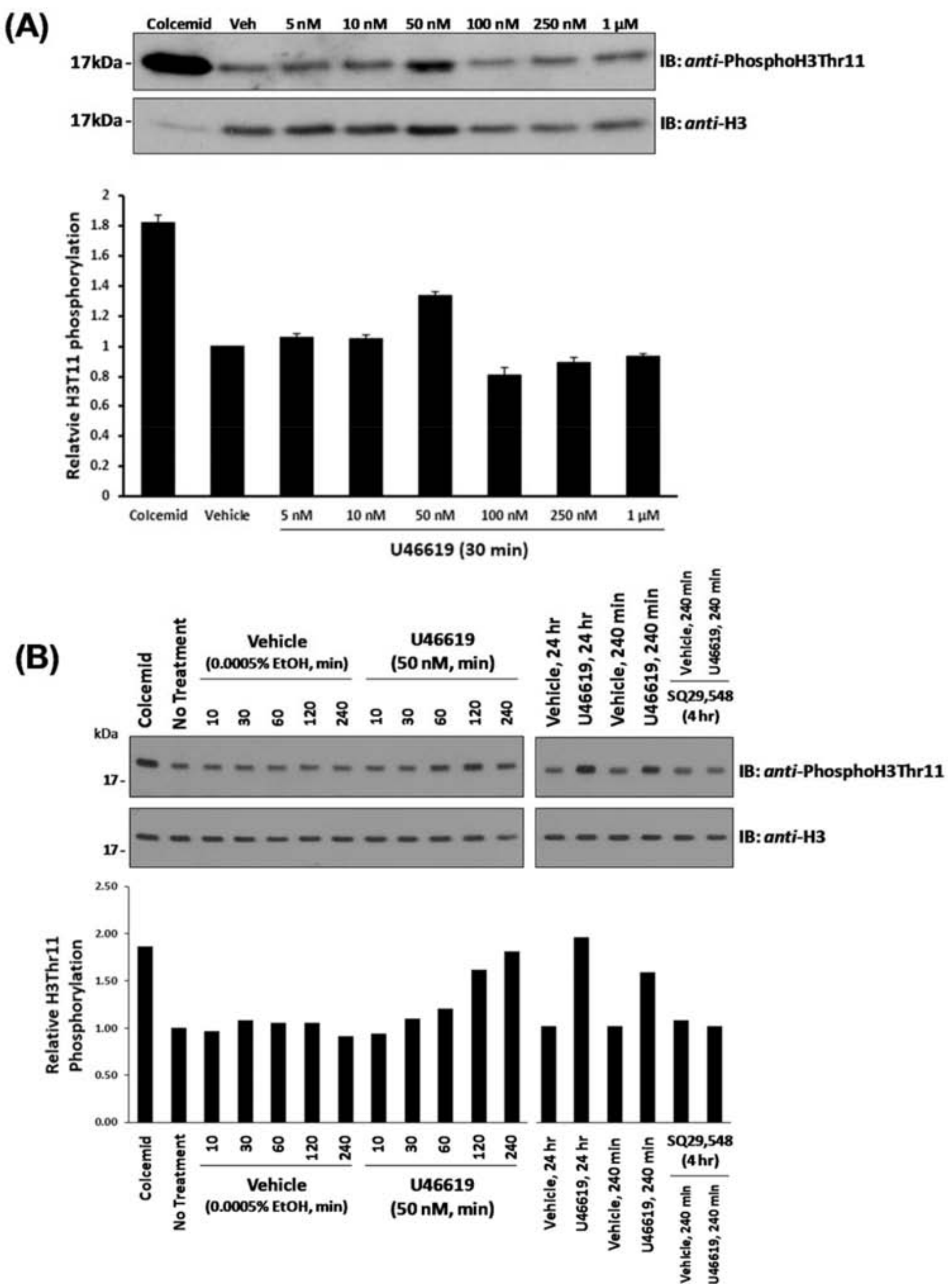

Supplementary Figure S7: Effect of TP agonist stimulation on H3Thr11 phosphorylation in PC-3 cells. Panel **A:** H3Thr11 phosphorylation in PC-3 cells following stimulation with U46619 (5 nM - 1 µM) or vehicle (0.0005% EtOH) for 30 min. As a positive reference for H3Thr11 phosphorylation, cells were growth-arrested with colcemid (50 ng/ml) for 24 hr. Panel **B.** H3Thr11 phosphorylation in PC-3 cells following stimulation with U46619 (50 nM), SQ29548 (1 µM), or U46619 (50 nM) plus SQ29548 (1 µM) or vehicle (0.0005% EtOH) for 10, 30, 60, 120, 240 min or 24 hr, as indicated. *Panels A & B*: In all cases, extracted histones were immunoblotted (IB) with *anti*-phospho-H3Thr11 (upper panels) and back-blotted with *anti*-histone H3 (lower panels) antisera. The bar charts show the mean relative levels of H3Thr11 phosphorylation relative to total histone H3 levels, as determined by densitometry, where levels in the vehicle-treated cells are assigned *a* value of 1.

Supplementary Table S1. List of PCR Amplification Primers and Mutator Primers used for Site-Directed Mutagenesis

Plasmid Generated	Template	Oligonucleotide Primers (*/**)
pCMVTag2B:PRK2	pEGFP-C1:PRK2	5′: GAGA*GGATCC*ATGGCGTCCAACCCCGAAC<br>3′: GAGA*AAGCTT*TTAACACCAATCAGCAATGTAGTC
pCMVTag2B:PRK3	pOTB7:PRK3	5′: GAGA*GAATTC*ATGGAGGAGGGGGCGCCGCG<br>3′: GAGA*AAGCTT*TCAGGGTTCCAGGAATCGCTCTG
pCMVTag2b:PRK1^{T774A}	pCMVTag2B:PRK1	GGGGACCGGACCAGC**G**CATTCTGTGGGACCC
pCMVTag2b:PRK2$^{1–362}$	pCMVTag2B:PRK2	CCTGGTTGGAGTCCAAGT**TGA**ACCAGATCATCTTTCATGAGC
pCMVTag2b:PRK2$^{1–621}$	pCMVTag2B:PRK2	GTATACTTCCAAAATCTCAAT**GA**GAATACAAGCCT<br>GATACTCCTCAG
pCMVTag2b:PRK2$^{577–984}$	pCMVTag2B:PRK2	5′: GAGA*GGATCC*GCCCCACCACGAGCTTCTTCTCTTGG<br>3′: GAGA*AAGCTT*TTAACACCAATCAGCAATGTAGTC
pCMVTag2b:PRK2^{K686E}	pCMVTag2B:PRK2	CACAAATGAGATGTTTGCTATA**G**A**G**GCCTTAAAGA<br>AAGGAGATATTGTGG
pCMVTag2b:PRK2^{T816A}	pCMVTag2B:PRK2	GATATGGAGATAGAACAAGC**G**CATTTTGTGGCACTCCTG
pCMVTag2b:PRK3$^{1–309}$	pCMVTag2B:PRK3	GCCAGCAGCCCCTCC**T**AGGGCTGGCTTCGGACC
pCMVTag2b:PRK3$^{1–539}$	pCMVTag2B:PRK3	CATATGGAGCCTAGGACT**T**GACGTGGGCCATCTCCACCAGCC
pCMVTag2b:PRK3$^{512–889}$	pCMVTag2B:PRK3	5′: GAGA*GAATTC*AAGCCCCCACGCCTCTACCTCCCCCAG3′:<br>GAGA*AAGCTT*TCAGGGTTCCAGGAATCGCTCTG
pCMVTag2b:PRK3^{K588E}	pCMVTag2B:PRK3	GGGAAATACTACGCCATC**G**A**G**GCACTGAAGAAGCAGGAGG
pCMVTag2b:PRK3^{T718A}	pCMVTag2B:PRK3	GGGGACCGGACTAGC**G**CCTTCTGTGGCACCC

The sequences of all the specific primers used to generate the expression plasmids within this manuscript are listed. (*) Sub-cloning primer pair (5′/3′) sequences are shown 5′ → 3′ and the cloning sites are underlined in italics. (**) Site-directed mutagenesis primer sequences (sense mutator only) are shown 5′ → 3′, where the mutated nucleotides are underlined in bold, and the sequence of the antisense primer is inferred.
